# Ryanodine receptors are part of the myospryn complex in cardiac muscle

**DOI:** 10.1038/s41598-017-06395-6

**Published:** 2017-07-24

**Authors:** Matthew A. Benson, Caroline L. Tinsley, Adrian J. Waite, Francesca A. Carlisle, Steve M. M. Sweet, Elisabeth Ehler, Christopher H. George, F. Anthony Lai, Enca Martin-Rendon, Derek J. Blake

**Affiliations:** 1 Wellcome Trust Centre for Human Genetics, University of Oxford, Oxford, UK; 2 Division of Psychological Medicine and Clinical Neurosciences, Cardiff University, Cardiff, UK; 3 Genome Damage and Stability Centre, University of Sussex, Brighton, UK; 4 Randall Division for Cell and Molecular Biophysics, King’s College London, London, UK; 5 Institute of Molecular and Experimental Medicine, Wales Heart Research Institute, Cardiff University, Cardiff, UK; 6 Division of Biomedicine, School of Biosciences, Cardiff University, Cardiff, UK; 7 Nuffield Division of Clinical Laboratory Sciences, Radcliffe Department of Medicine, University of Oxford, Oxford, UK

## Abstract

The *Cardiomyopathy–associated gene 5* (*Cmya5*) encodes myospryn, a large tripartite motif (TRIM)-related protein found predominantly in cardiac and skeletal muscle. *Cmya5* is an expression biomarker for a number of diseases affecting striated muscle and may also be a schizophrenia risk gene. To further understand the function of myospryn in striated muscle, we searched for additional myospryn paralogs. Here we identify a novel muscle-expressed TRIM-related protein minispryn, encoded by *Fsd2*, that has extensive sequence similarity with the C-terminus of myospryn. *Cmya5* and *Fsd2* appear to have originated by a chromosomal duplication and are found within evolutionarily-conserved gene clusters on different chromosomes. Using immunoaffinity purification and mass spectrometry we show that minispryn co-purifies with myospryn and the major cardiac ryanodine receptor (RyR2) from heart. Accordingly, myospryn, minispryn and RyR2 co-localise at the junctional sarcoplasmic reticulum of isolated cardiomyocytes. Myospryn redistributes RyR2 into clusters when co-expressed in heterologous cells whereas minispryn lacks this activity. Together these data suggest a novel role for the myospryn complex in the assembly of ryanodine receptor clusters in striated muscle.

## Introduction

The unique cytoskeletal organisation of striated muscle is dependent upon the formation of specialised interactions between proteins that have both structural and signalling functions^1^. For example, the sarcomere is organised around the 4 MDa protein titin; the largest protein in the vertebrate proteome, that spans half the length of the sarcomere from the Z-disc to the M-line. The Z-disc provides the framework for the assembly and anchoring of the numerous multi-protein complexes of the sarcomere and also integrates signal transduction events that are thought to mediate the stretch response^2, 3^. In addition to titin, striated muscle expresses a repertoire of very large proteins that interact with the unique structural elements of the sarcomere. Many of these enigmatic proteins including obscurin, nebulin and titin have a repeating, modular architecture and interact with a myriad of different proteins in the myocyte and cardiomyocyte^4–8^.

Myospryn, encoded by the *CMYA5* gene, is a similarly enigmatic 500 kDa protein that is expressed predominantly in skeletal and cardiac muscle^9, 10^. Myospryn has greatest sequence similarity to the midline E3 ubiquitin ligases that are members of the TRIM superfamily of proteins and has also been designated TRIM76^9, 11, 12^. This similarity does not encompass the RING domain of the midline proteins potentially negating the possibility that myospryn is an E3 ubiquitin ligase. While the function of myospryn in muscle is unknown, levels of its transcript are altered in diseases affecting striated muscle including Duchenne muscular dystrophy (DMD), experimentally-induced hypertrophy and cardiomyopathy^13–16^. Furthermore, heterozygous *CMYA5* mutations have recently been identified by whole exome sequencing in Chinese patients with sporadic hypertrophic cardiomyopathy whereas *CMYA5* polymorphisms are associated with left ventricular wall thickness in patients with hypertension^17, 18^. Myospryn expression is positively regulated *in vivo* by the transcription factor Mef2a and is consequently down-regulated in *Mef2a*-deficient mice^10^. In addition to muscle disease, common *CMYA5* genetic variants have also been associated with an increased risk of schizophrenia^19, 20^.

Myospryn was originally identified in a yeast two-hybrid screen for proteins interacting with dysbindin; a component of the biogenesis of lysosome-related organelles complex-1 (BLOC-1) endosomal trafficking complex^9^. In addition to dysbindin, myospryn has been shown to interact with desmin^21^, α-actinin^10^, the RIIα regulatory subunit of PKA^22, 23^, dystrophin^22^, calcineurin^24^, titin and calpain-3^25^. In spite of myospryn’s size, all of the currently identified myospryn interactors bind to overlapping regions in the C-terminus of the protein encompassing the tripartite motif^15, 25, 26^. For example, α-actinin, the regulatory subunit of protein kinase A (PKA, RIIα) and titin bind to overlapping sites within the C-terminus of myospryn that include the FN3 domains, the BBC domain and the SPRY domain^10, 23, 25, 27^. Similarly, calpain-3 and dysbindin bind to the region encompassing the BBOX’/BBC and first FN3 domains whereas desmin binds to the distal part of the SPRY domain at the extreme C-terminus of myospryn^9, 21, 25^.

Given the number of myospryn-binding proteins described to date, it is not surprising that its specific function(s) in striated muscle remains unresolved. Furthermore, the subcellular location of myospryn in muscle is far from clear. Myospryn has been shown to co-localise with α-actinin, dystrophin and the RIIα regulatory subunit of PKA at the costameres of skeletal muscle^10, 22, 23^. It has therefore been suggested that myospryn may be a costameric A-kinase anchoring protein^22, 23^. By contrast, Sarparanta and colleagues showed that myospryn immunoreactivity flanked the Z-line and was also present at sites along the sarcomere including the M-band where it can interact with titin and calpain-3^25^. In cardiac muscle myospryn is reported to be found predominantly at the intercalated discs and costameres co-localising with desmin and, at the perinuclear region and endoplasmic/sarcoplasmic reticulum of cultured neonatal cardiomyocytes^21^.

To gain further insight into the function of myospryn in striated muscle we used a combination of biochemical and immunochemical techniques to find physiologically relevant myospryn-associated proteins. Using immunoaffinity chromatography and mass spectrometry we show that myospryn exists as part of an oligomeric protein complex in cardiac muscle with the cardiac ryanodine receptor (RyR2). This complex also contains a previously unreported paralog of myospryn that we have named minispryn.

## Results

### Cloning and initial characterisation of minispryn

Previously we reported that myospryn has a BBOX’ domain required for binding to dysbindin^9^. To find other proteins that have a similar architecture, we searched the NCBI (National Center for Biotechnology Information) databases for myospryn paralogs that contained this sequence. This search identified a single expressed sequence tag (EST, srf30) that was predicted to encode a BBOX’-containing protein^9^. A PCR product spanning the EST was used to screen a skeletal muscle cDNA library for full-length cDNA clones, thus yielding mini7E. The full-length sequence of mini7E is 6148 bp and has a single large open reading frame that encodes a 716 amino acid protein with a predicted molecular mass of 81 kDa (Supplementary Fig. S1). Based upon its size and paralogy with myospryn (see below), we have named this protein minispryn. The NCBI annotation for the genes encoding minispryn and myospryn are *Fsd2* (fibronectin type III and SPRY domain containing 2, Gene ID: 244091) and *Cmya5* (Gene ID: 76469) respectively. For convenience, in this manuscript we have used the names *Fsd2* and *Cmya5* to describe the gene, and minispryn and myospryn to describe their respective cognate proteins.

**Figure 1:**
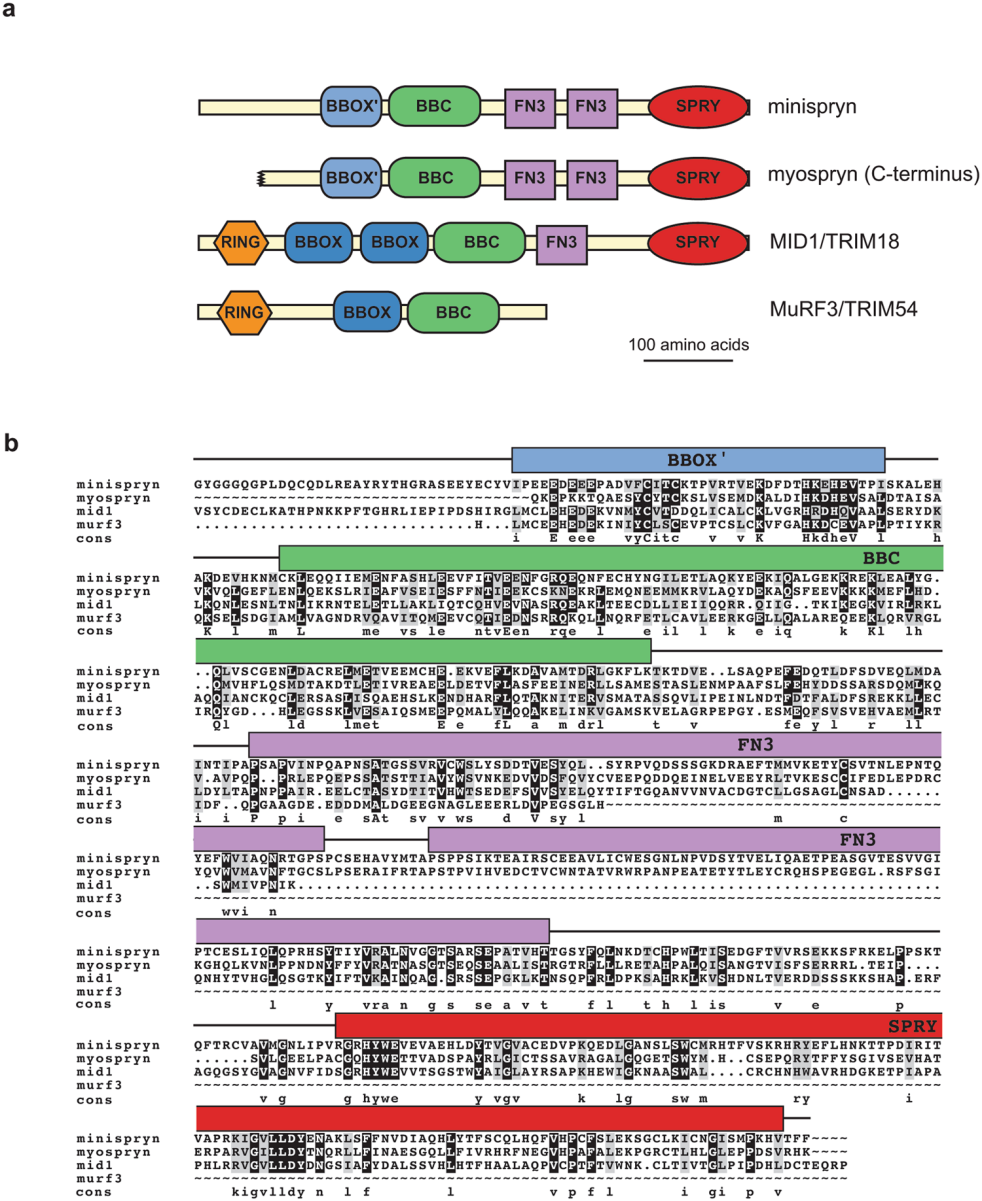
Minispryn bioinformatics. (**a**) Domain architecture of minispryn and related proteins. Minispryn, myospryn and two members of the TRIM-protein family share extensive sequence similarity at their C-termini. While the TRIM proteins are E3 ubiquitin ligases, the absence of the RING domain in minispryn and myospryn probably preclude this function. (**b**) Multiple sequence alignment. The complete coding sequence of minispryn aligns with amino acids 3171–3739 of myospryn (CAE02649.1), 139–706 of midline-1 (XP_017173902) and 123–366 of murf3 (NP_067422.1) spanning the BBOX/BBOX’ and BBC domains of each protein and the FN3 and SPRY domains of myospryn and midline-1.

Minispryn shares 45.5% protein sequence similarity with the C-terminus of myospryn retaining the same domain architecture and order; BBOX’, BBC, two FN3 domains and a C-terminal SPRY domain (Fig. 1a). By contrast, minispryn lacks the large N-terminal region that constitutes the majority of the myospryn protein including the low complexity repeat region^9^. Other than myospryn, the closest vertebrate minispryn homologs are the midline proteins encoded by the *MID1* and *MID2* genes and the Muscle Ring Finger (MuRF) proteins (Fig. 1b). The midline and MuRF proteins contain the canonical RING, BBOX and BBC domains found in the majority of the TRIM family of E3-ubiquitin ligases^11^. By contrast, minispryn and myospryn only contain the C-terminal domains that follow the BBOX of the midline proteins and are thus devoid of an apparent RING-domain (Fig. 1b). Furthermore, minispryn and myospryn have tandem FN3 domains whereas the midline proteins only have a single FN3 domain.

Comparison of the genomic organization of *Fsd2* and *Cmya5* shows that the architecture of each gene is remarkably similar, both being composed of 13 exons (Supplementary Fig. S2A). The difference in size of the two proteins is attributable to the sequence encoded by exon 2, which in *Cmya5* is 9.3 kb but only 582 bp in *Fsd2*. Long-range sequence analysis around the CMYA5 and FSD2 loci showed that each gene is contained within a conserved chromosomal duplication (Supplementary Fig. S2B). The *CMYA5* cluster that contains the *HOMER1*, *JMY* and *AP3B1* genes spans approximately 2.5 Mbp of chromosome 5 whereas the *FSD2* cluster on chromosome 15 that contains *HOMER2*, WHAMM (*JMY* paralog) and *AP3B2* spans only 240 kb. The genomic architecture of the *CMYA5* and *FSD2* clusters is also conserved in teleost fish species such as *Tetraodon* that diverged from a common ancestor approximately 450 million years ago.

### Minispryn transcript and protein

**Figure 2:**
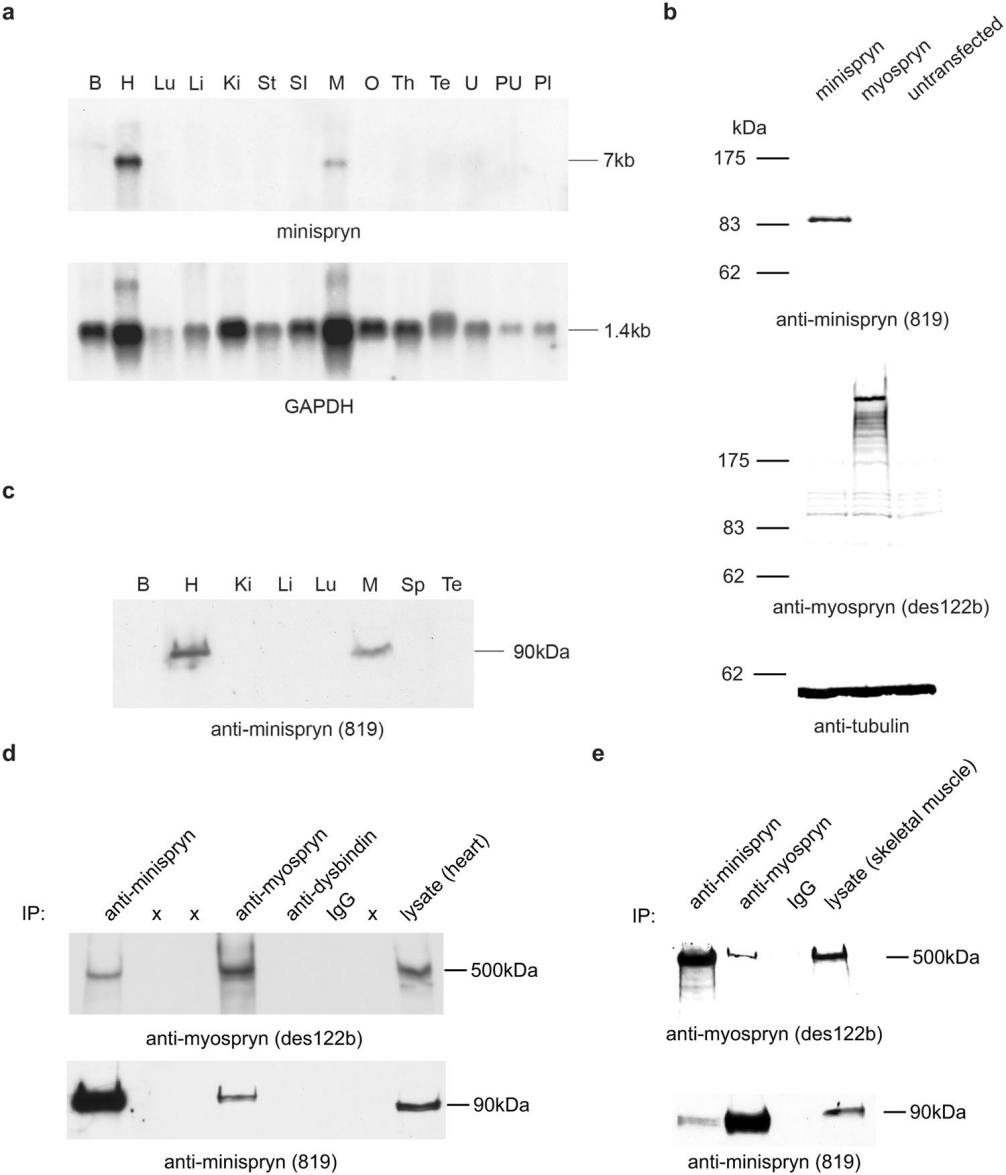
Minispryn transcript and protein. (**a**) Northern blot showing the size and tissue distribution of the murine minispryn transcript. A single band of approximately 7 kb is only detected in heart and skeletal muscle. The blot was stripped and re-hybridized with a GAPDH probe to demonstrate the presence of mRNA in each lane. Key: B, brain; H, heart; Lu, lung; Li, liver; Ki, kidney; St, stomach; SI, small intestine; M, muscle; O, ovary; Th, thymus; Te, testes; U, uterus; PU, pregnant uterus; Pl, placenta; Sp, spleen. (**b**) Western blot of several mouse tissues showing that minispryn is a protein of approximately 90 kDa that is found predominantly in heart and skeletal muscle. (**c**) Antibody specificities were determined in heterologous cells. Lysates prepared from HEK-293T cells expressing full-length myospryn (clone 21B) and minispryn (clone 7E) were processed for immunoblotting with antibodies detecting their cognate antigen. The 819 antibody only detects minispryn in transfected cells whereas des122 only detects myospryn under the same conditions. The lysates were also immunoblotted with anti-tubulin antibodies to demonstrate similar levels of protein in each transfection. Lysates prepared from untransfected HEK-293T cells are also shown. (**d**) In tissue, minispryn and myospryn were immunoprecipitated from RIPA extracts of cardiac muscle using specific antibodies. Minispryn and myospryn are robustly immunoprecipitated with their cognate antisera but also co-IP with the antisera raised against the paralogous protein. Neither myospryn nor minispryn are immunoprecipitated with unrelated IgG. (**e**) Minispryn and myospryn similarly co-immunoprecipitate from RIPA extracts of skeletal muscle. X denotes an empty lane.

Northern blot analysis shows that the 7 kb minispryn transcript is expressed at highest levels in heart but is also detectable in skeletal muscle (Fig. 2a). An anti-minispryn antibody (819) was raised in rabbits against the first 227 amino acids of minispryn. The specificity of the anti-myospryn and anti-minispryn antibodies was determined by immunoblotting extracts prepared from HEK293T cells over-expressing untagged myospryn and minispryn. Each antibody detects its cognate antigen and does not cross-react with their respective paralog (Fig. 2b). The tissue distribution of minispryn in the adult mouse was confirmed with the anti-minispryn antibody that detects a protein of approximately 90 kDa in cardiac and skeletal muscle (Fig. 2c). We used publically available expression data to examine the relative abundance of the transcripts encoding minispryn and myospryn in heart and skeletal muscle^28^. *FSD2* was expressed at relatively low levels in heart (6.3 RPKM (Reads Per Kb of transcript per Million mapped reads)) and skeletal muscle (5.6 RPKM). By contrast, levels of *CMYA5* where significantly higher in skeletal muscle (388.3 RPKM) compared to heart (94.2 RPKM). Interestingly, these data place *CMYA5* (myospryn) in the top 100 most highly expressed transcripts in skeletal muscle. The relative abundance of each transcript is mirrored by the levels of the cognate protein in the same tissue (Fig. 2c)^9^.

Since paralogous proteins often participate in similar protein:protein interactions we used co-immunoprecipitation to determine whether minispryn and myospryn form a protein complex *in vivo*. Proteins were co-immunoprecipitated from heart and skeletal muscle RIPA extracts using myospryn- and minispryn-specific antibodies. Myospryn and minispryn were robustly immunoprecipitated with their cognate antisera (Fig. 2d and e). Importantly, minispryn was detected in the protein complex immunoprecipitated with anti-myospryn antisera. Conversely, myospryn was detected in material immunoprecipitated by the anti-minispryn antibody. In control experiments, neither myospryn nor minispryn could be immunoprecipitated from cardiac or skeletal muscle with an unrelated IgG (Fig. 2d and e).

### Immunoaffinity purification of the myospryn complex from cardiac muscle

**Figure 3:**
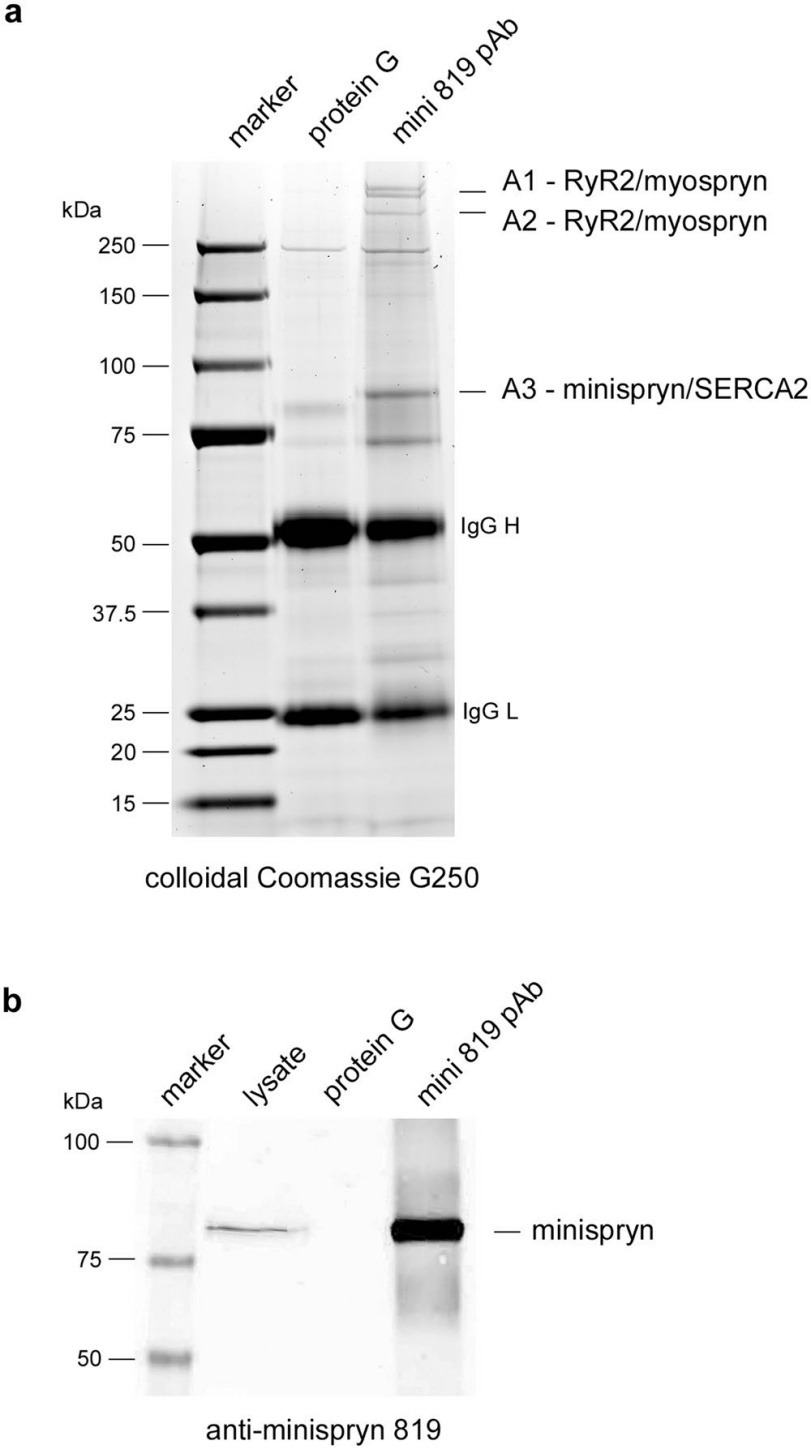
Immunoaffinity purification of the myospryn complex from cardiac muscle. (**a**) Proteins eluted from an anti-minispryn immunoaffinity column or protein G only column were separated by PAGE and stained with colloidal Coomassie blue dye. Bands A1-A3 that were only present in minispryn immunoaffinity eluate were excised and processed for mass spectrometry. The identity of the major protein(s) found in each band is listed. Note that in this study we focussed exclusively on proteins in bands A1, A2 and A3. Corresponding peptides for myospryn, RyR2 and minispryn are shown in Supplementary Dataset 1. (**b**) Robust minispryn enrichment following immunoaffinity purification was demonstrated by immunoblotting using the 819 anti-minispryn antibody. The sizes of the molecular weight markers are shown in kDa.

To search for additional minispryn:myospryn associated proteins, we used the 819 antibody to immunoaffinity purify minispryn and associated proteins from mouse heart. Immunoprecipitated proteins were eluted from the immunoaffinity matrix and resolved on Novex gradient gels stained with colloidal Coomassie blue (Fig. 3a). Minispryn (band A3) was highly enriched following immunoaffinity purification demonstrating the efficacy of this technique for protein purification from tissue (Fig. 3b). Bands that were not shared with the protein G control were excised from the gel, digested with trypsin and analysed by mass spectrometry. Tryptic peptides derived from minispryn (band A3) and myospryn (band A1 and A2) were readily identified, supporting our finding that both proteins exist in a complex *in vivo* (Table 1). Furthermore, numerous peptides corresponding to the cardiac ryanodine receptor (RyR2) were obtained from band A1 at approximately 550 kDa. Although SERCA2 was also identified in band A3, we have not confirmed this potential interaction experimentally (Fig. 3a). In control experiments, myospryn was immunoaffinity purified from cardiac muscle using des122-conjugated agarose beads (Supplementary Fig. S4). Once again, bands were excised and processed for mass spectrometry as described above. Tryptic peptides derived from myospryn, minispryn and RyR2 were identified in the myospryn immunoaffinity purification (Supplementary Dataset 1). Interestingly, previously described interactors such as α-actinin did not co-purify with myospryn under the conditions used to immunoaffinity purify the complex from cardiac muscle (Supplementary Fig. S4)^10^.

**Table 1:** Mass spectrometry statistics for minispryn and myospryn immunoaffinity purification from mouse heart.

Protein		Score	Coverage	Peptides	MW
**MINISPRYN IAP**	ryanodine receptor 2, cardiac [Mus musculus]/gi124430578 (A1)	1496.39	24.16	120	564.5
	stretch-responsive fibronectin protein type 3 [Mus musculus]/gi45774102 (A1)	1458.93	34.30	110	397.5
	fibronectin type III and SPRY domain-containing protein 2 [Mus musculus]/gi116063550 (A3)	432.45	36.31	31	81.4
**MYOSPRYN IAP**	ryanodine receptor 2, cardiac [Mus musculus]/gi124430578	3427.78	36.57	246	564.5
	stretch-responsive fibronectin protein type 3 [Mus musculus]/gi45774102	3008.80	42.70	188	397.5
	fibronectin type III and SPRY domain-containing protein 2 [Mus musculus]/gi116063550	463.66	36.73	58	81.4

Proteins identified by mass spectrometry from minispryn (Fig. 3a) and myospryn (Supplementary Fig. S4) immunoaffinity purification from cardiac muscle. For clarity, in this study we have only focussed on the three most significant matches from each experiment. Note that stretch-responsive fibronectin protein type 3 [*Mus musculus*]/gi45774102 is the alternative name for *Cmya5*/myospryn and fibronectin type III and SPRY domain-containing protein 2 [*Mus musculus*]/gi116063550 is *Fsd2*/minispryn.

### Immunolocalisation of myospryn in striated muscle

**Figure 4:**
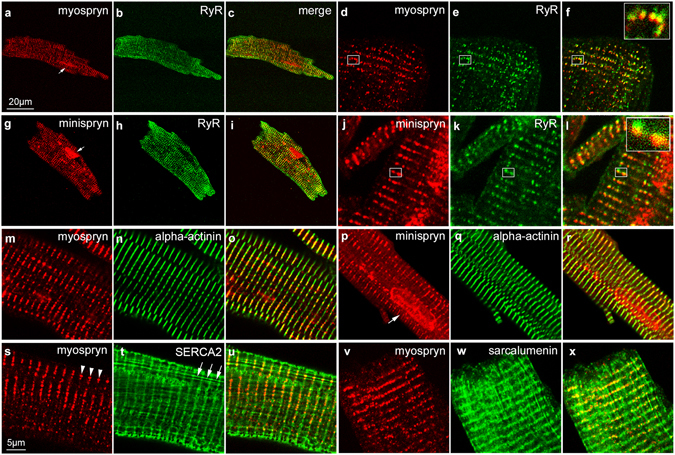
Immunolocalization of myospryn and minispryn in isolated cardiomyocytes. Enzymatically isolated guinea pig ventricular cardiomyocytes were fixed and processed for confocal immunofluorescence microscopy using the indicated antibodies. (**a**–**c**) At low magnification myospryn (a) immunoreactivity is visible in discontinuous striations that run perpendicular to the long axis of the cardiomyocyte partially overlapping the nucleus (arrow in a). (**d**–**f**) Higher magnification images show that myospryn immunoreactivity is present in distinct punctae that co-localise with ryanodine receptors (e) in the SR (f and inset). (**g**–**i**) Anti-minispryn (g) immunoreactivity also appears in discontinuous striations running the length of the cardiomyocyte and is also present in the nucleus (arrow in g). (**j**–**l**) In common with myospryn, higher magnification images show that minispryn partially co-localises with ryanodine receptors (k) in the SR (l and inset). (**m–o**) Myospryn immunoreactivity overlaps the Z-line labelled with an anti-alpha-actinin antibody (m, n, o). (**p**–**r**) Similarly, minispryn immunoreactivity also partially overlaps alpha-actinin at the Z-line (p, q, r). (**s**–**x**) Cardiomyocytes labelled with either anti-SERCA2 (s, t, u) or anti-sarcalumenin (v, w, x) antibodies and myospryn antibodies demonstrate that myospryn immunoreactivity (arrowheads in s) appears to be restricted to the j-SR rather than the l-SR (arrows in t).

Confocal microscopy was used to localise myospryn and minispryn in isolated adult guinea pig ventricular cardiomyocytes. At low magnification, myospryn immunoreactivity was concentrated in discontinuous stripes that ran tangentially to the long axis of the cardiomyocyte (Fig. 4a and d). Myospryn immunoreactivity was also present diffusely throughout the myocyte and also in or around the nucleus as described previously (arrow in Fig. 4a)^21^. A similar pattern of immunoreactivity was found with the anti-RyR antibody within the same cell suggesting that myospryn is localised to the sarcoplasmic reticulum (SR) of cardiomyocytes (Fig. 4b). At higher magnification, myospryn and the RyRs co-localise in distinct punctae throughout the cell (Fig. 4d–f). Similarly, minispryn immunoreactivity was detected in discontinuous striations running perpendicular to the long axis of the cardiomyocyte that partially co-localise with RyR2 (Fig. 4g–j). Punctate myospryn immunoreactivity also overlapped with the continuous α-actinin labelling at the Z-line of the cardiomyocyte (Fig. 4m–o). Minispryn immunoreactivity also overlapped α-actinin at the Z-line (Fig. 4p–r) and despite the absence of an apparent nuclear localisation signal, minispryn was also found in or around the nucleus (arrow in Fig. 4g and p). Double labelling with antibodies raised against SERCA2 (Fig. 4s–u) or sarcalumenin (Fig. 4v–x) showed that myospryn is associated with the junctional SR (j-SR) rather than the majority of the longitudinal SR (l-SR) that also contain SERCA2 and sarcalumenin (Fig. 4s–x).

We also examined the subcellular localisation of myospryn and minispryn in skeletal muscle using antibodies that label different compartments of the myocyte (Supplementary Fig. S6). In longitudinal sections of guinea pig quadriceps muscle, minispryn is localised to a region flanking the Z-line, labelled with an anti-α-actinin antibody (Supplementary Fig. S6). No labelling of the sarcolemma was observed. By contrast to α-actinin labelling, minispryn immunoreactivity is concentrated in small punctae that run parallel to the Z-line. This pattern is reminiscent of the sarcoplasmic reticulum as shown by double immunofluorescence labelling of minispryn and the RyRs in the same section. Both antigens have a similar and partially overlapping distribution in longitudinal sections suggesting that minispryn also concentrates at the SR. In skeletal muscle myospryn (and minispryn) punctate immunoreactivity is observed flanking the Z-line (at the A-I band interface) in close apposition to RyRs whereas in cardiomyocytes, the terminal cisternae of the SR are localised directly above the Z-line in fixed and live cardiomyocytes (Fig. 4 and Supplementary Fig. S6)^29^. Thus, the differences in myospryn immunolocalisation between cardiac and skeletal muscle are consistent with the differential geometry of the SR characteristic of the two types of striated muscle^30, 31^.

### Myospryn clusters RyR2 in heterologous cells

**Figure 5:**
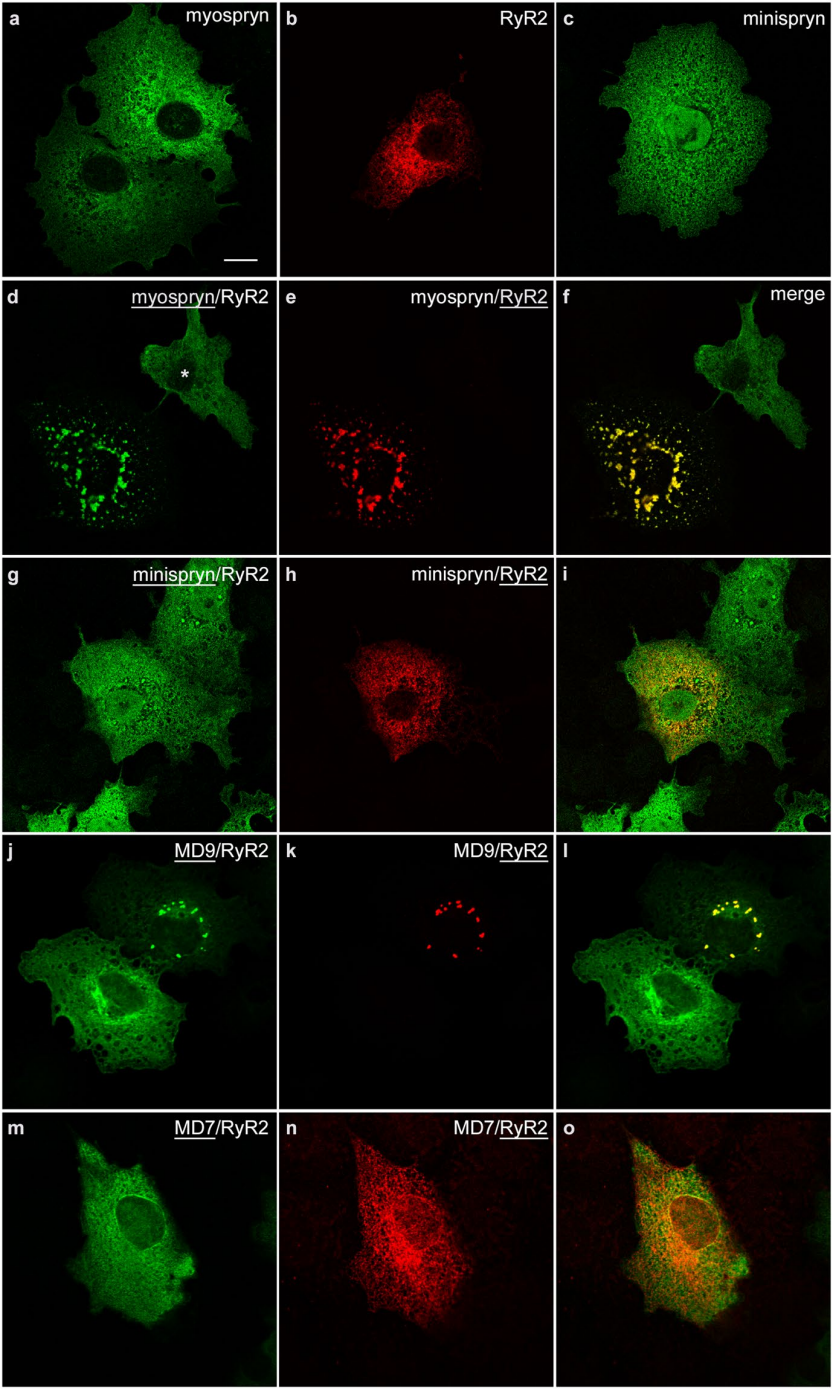
Myospryn clusters RyR2 in heterologous cells. COS-7 cells were transfected with expression constructs as indicated. (**a**–**c**) Full-length myospryn (a) and minispryn (c) appear to be associated with the internal membranes of the cell when expressed on their own whereas RyR2 (b) labelling is restricted to the endoplasmic reticulum. (**d**–**f**) Co-expression of myospryn (d) and RyR2 (e) results in the clustering of both proteins in intensely staining punctae (merged image, f). Note that myospryn is not clustered in the adjacent cell (asterisk) that does express RyR2. (**g**–**i**) By contrast co-expression of minispryn (g) and RyR2 (h) does not alter the distribution of either protein (merged image, i). (**j** *–* **o**) Truncated myospryn constructs were used to delineate the regions of the protein involved in RyR2 clustering. MD9 (j) was able to cluster RyR2 (k and merged image, l) whereas the smaller protein MD7 (m) failed to cluster the receptor (n and merged image, o). The underlining denotes the transfected construct for each channel. Scale bar = 10 µm.

To explore the functional significance of the association between myospryn, minispryn and RyR2, we expressed different combinations of each cDNA in COS-7 cells. In transfected cells expressing only one construct, we found that full length myospryn and minispryn were distributed throughout the cytoplasm of the cell and appeared to associate with intracellular membranes (Fig. 5a and c). We also observed that minispryn immunoreactivity was found in the nucleus whereas myospryn was apparently excluded. By contrast, RyR2 was found in the endoplasmic reticulum as expected (Fig. 5b). It is noteworthy that endogenous myospryn, minispryn and RyR2 were not detected in COS-7 cells with the antibodies used in this study. Co-expression of myospryn with RyR2 caused the dramatic relocalisation of both proteins into intensely staining punctae (Fig. 5d–f). By contrast, co-expression of minispryn with RyR2 had no apparent effect on the localisation of either protein (Fig. 5g–i). Co-expression of myospryn and minispryn in COS7 cells does not appear to cause the redistribution of either protein. We also used a range of truncated myospryn constructs to map the region of the protein that clusters RyR2^9, 25^. MD7 (amino acids 3039–3739) that contains the BBOX’, BBC, FN3 and SPRY domains was unable to cluster RyR2. By contrast, the larger construct MD9 (amino acids 2731–3739) that contains all of the aforementioned C-terminal domains and an additional contiguous 311 amino acids efficiently clusters RyR2 in cells expressing both constructs (Fig. 5j–l). These data indicate that a conserved region encompassing amino acids 2731–3041 of mouse myospryn is likely to be required for RyR2 clustering. Interestingly, this region is not found in minispryn because the region of sequence similarity only extends from amino acids 3179 to 3736 of myospryn^9^. Furthermore, the RyR2-clustering region of myospryn is in an evolutionarily conserved region of the protein that is distinct from the other ligand binding sites in the TRIM-like domain^15, 26^.

## Discussion

The cytoarchitecture of striated muscle is governed in part by the assembly of macromolecular protein complexes that form connections between the myofilaments in the sarcomere and sarcolemma of the myocyte. In order to do this, the myocyte produces a number of unusually large modular proteins that integrate mechanical and signalling processes throughout the cell^7, 8^. Electrical excitation of the myocyte leads, via Ca^2+^-signalling, to activation of the myofilament and contraction. Electrical activity is propagated throughout the myocyte by branched invaginations of the sarcolemma known as transverse tubules (t-tubules) that form a highly connected network of tubules that surround myofilaments^32^. T-tubules are involved in EC-coupling by concentrating voltage-gated Ca^2+^ channels (VGCC) such as the dihydropyridine receptor in close proximity to the RyRs in the j-SR^33^. The precise juxtaposition of the VGCC with Ca^2+^-releasing RyRs ensures tight coupling between electrical activity and Ca^2+^-signalling at so-called couplons; dyads in cardiac muscle and triads in skeletal muscle^34–36^. Myospryn, one the largest proteins in striated muscle, has been ascribed a number of structural and signalling functions mediated by interactions with different components of the myocyte^15, 26^. In this paper, we have used immunoaffinity purification and mass spectrometry to show that myospryn is part of a multiprotein complex in cardiac muscle that contains a novel myospryn paralog, minispryn and the cardiac ryanodine receptor, RyR2.

To gain further insight into the role of minispryn and myospryn we use a panel of antibodies to immunoaffinity purify each protein directly from cardiac and skeletal muscle. Using a combination of mass spectrometry and western blotting, we show that myospryn and minispryn co-immunoprecipitate from both tissues and are found in a complex with RyR2 in cardiac muscle (Fig. 3a). Importantly, these data were also confirmed when RyRs were immunoaffinity-purified from heart using the well-characterised pan-RyR antibody, 34C^37^. It is also noteworthy that the myospryn interactors, PKA, RIIα and calcineurin have all been shown to interact with RyR2^38, 39^. Furthermore, myospryn and minispryn co-localise with RyRs in isolated adult ventricular cardiomyocytes (Fig. 4). Since myospryn and minispryn lack predicted transmembrane domains, our data suggest that the myospryn complex is concentrated at dyad couplons in cardiac muscle. Similarly, myospryn and minispryn also co-localise with skeletal muscle RyRs at the j-SR (Supplementary Fig. S6). In common with cardiac muscle, the SR of striated muscle can be divided into two sub-domains; the j-SR or triad couplon formed between the t-tubule and terminal cisternae of the SR and the l-SR^30, 31, 35, 40^.

To our surprise, we found that myospryn was able to cluster RyR2 in heterologous cells (Fig. 5). Conserved regions in the C-terminus of myospryn that are not present in minispryn mediated RyR2 clustering. To the best of our knowledge this is the first example of a protein that can actively cluster RyRs. However, in striated muscle it is also possible that myospryn promotes the apparent clustering of RyRs by altering the structure of the SR. It is therefore tempting to speculate that one function of the myospryn complex in cardiac muscle is in the formation and maintenance of RyR clusters. Although it is clear that RyR clustering is essential for EC coupling, little is known about the processes driving receptor aggregation and the role of components of the couplon in this process^41^. Expression of RyRs in heterologous cells results in distribution of the protein throughout the ER. Indeed, biophysical studies on the inositol 1,4,5-trisphosphate receptor (IP_3_R) and RyR1 expressed in COS7 cells show both proteins have similar diffusion rates in the endoplasmic reticulum indicative of free movement through the subcellular domain^42^. By contrast, RyRs extracted from skeletal muscle and heart tissue can self-associate into discrete, higher order configurations^43, 44^.

Weakened calcium transients mediated by RyRs are one of the major pathological features underlying heart failure. Although there are well documented differences between the SR structure in skeletal and cardiac muscle, the maintenance of RyR clusters appears to be essential for physiological EC-coupling^45^. Maladaptive t-tubule remodelling in heart failure and hypertrophic cardiomyopathy leads to abnormalities in Ca^2+^-signalling associated with orphaned RyRs^46–48^. Orphaned RyRs appear to be spatially distinct having lost their couplings with the DHPRs in the t-tubule system. Recently, two-photon microscopy has shown that defects in t-tubular electrical activity are responsible for the abnormalities in Ca^2+^-signalling observed in different animal models of heart failure and hypertrophic cardiomyopathy^49, 50^. It would therefore be important to determine whether the myospryn complex is involved in the uncoupling of RyRs in different models of heart failure and cardiomyopathy.

It is well established that adult ventricular cardiomyocytes dedifferentiate in culture resulting in the dramatic loss of t-tubules, effectively precluding their use for acute knockdown experiments aim at studying the function of the myospryn complex *ex vivo* ^51, 52^. However, the physiological role of the myospryn complex in striated muscle could be determined using transgenic animals with mutations in *Cmya5* and *Fsd2*. If viable, RyR2 clustering and SR function in cardiomyocytes lacking myospryn and minispryn could be determined using state of the art imaging techniques as described recently^49^. Similarly, high resolution imaging and electron microscopy could be used to examine the association of the myospryn complex with other components of the cardiomyocyte using a panel of antibodies^53–55^. This may be particularly informative because most of the antibodies developed to date have been generated against epitopes in the extreme C-terminus of myospryn^9, 21, 25^. It is therefore possible that other regions of a conserved large protein like myospryn can interact with different subcellular components of the cardiomyocyte.

It is tempting to speculate that differential expression of *CMYA5* in cardiomyopathies and muscular dystrophy may contribute to the disease process through physical interactions with RyRs that affect their clustering or association with other proteins in the couplon. It is noteworthy that alterations in the post-transcriptional and translational regulation of the genes encoding numerous SR components converging on RyR2 have been documented in heart failure^56^. Interestingly, a common polymorphism in *CMYA5* (K2906N) has also been associated with left ventricular wall thickness in patients with hypertension^17^. This polymorphism is immediately proximal to the evolutionary conserved region in myospryn that is required for RyR2 clustering (Fig. 5). In addition, *CMYA5* and *RYR2* mutations have recently been found in patients with hypertrophic cardiomyopathy^18^. Whether these *CMYA5* and *RYR2* mutations are pathogenic or merely rare polymorphisms remains to be determined through replication in larger samples. Taken together, our data suggest a novel role for the myospryn complex in diseases of cardiac and skeletal muscle that is potentially mediated through interactions with protein components of the couplon. Our data provide a possible molecular basis for previous observational studies suggesting a role for myospryn in cardiomyopathy, muscle hypertrophy and muscular dystrophy^13–16^.

## Methods

### Molecular Biology

A 2.2 kb PCR product spanning the srf30 EST that encodes the BBOX’ domain of a hitherto unknown cDNA^9^ was produced from muscle first strand cDNAs using the primers, 5′-GACTCAGAACATCCACTGACTGAG and 5′-GGAAATACCAAAAGGGCCTAAAAG. This product was radiolabelled with ^32^P-dCTP and used to screen a murine adult muscle cDNA library constructed in pPC86 (Invitrogen) using standard methodology. Several positively-hybridising cDNA clones were identified and end-sequenced. The complete sequence of the longest cDNA clone, mini7E, was obtained. The entire open reading frame of minispryn was amplified by PCR with the primers 5′-GTCGACATGAGAGAGGAAGCAGAGGAG and 5′-GGATCCCTAAAAGAAAGTGACATGCTTTGG and cloned into the SalI/BamHI sites of pEGFP-C1 (Clontech) and fully sequenced to produce GFP-minispryn. Expression constructs encoding the C-terminus of myospryn (clone MD9 and MD7) and full length myospryn (clone c21B) have been described previously^9^. The murine RyR2 construct (mRyR2) was kindly supplied by Dr S. Wayne Chen^57^. Sequence alignments were performed using PILEUP (GCG version 10) and shaded with BOXSHADE (http://www.ch.embnet.org/software/BOX_form.html). Domains in myospryn and minispryn were identified using the program SMART (http://smart.embl-heidelberg.de/)^58^.

### Antibodies

Rabbit polyclonal antisera were raised against minispryn as follows. A PCR product encoding amino acids 1 to 227 of minispryn was produced by PCR using the primers, 5′-TGAATTCATGGAGGAGGAAGCAGAG and 5′-TGTCGACTCAATTGTAATGGCACTCAAAGTTTT. This region of minispryn has no sequence similarity to myospryn minimising any potential cross-reactivity between antibodies raised against the two proteins. The resulting PCR product was cloned downstream of thioredoxin in pET32a (Novagen) to produce a chimeric fusion protein that was used to immunize New Zealand white rabbits. Anti-thioredoxin antibodies were removed by affinity purification on a Sulfolink (Pierce) thioredoxin column and the resulting anti-minispryn antibody (819) was affinity-purified against the cognate antigen coupled to Sulfolink. The des122 anti-myospryn antibody was raised against amino acids 2791 to 2913 of murine myospryn in rabbits as described previously ^9^. The anti-α-actinin monoclonal antibody, clone EA-53, was purchased from Sigma. The 34C (anti-ryanodine receptor) and XIIC4 (anti-sarcalumenin) monoclonal antibodies were obtained from the Developmental Studies Hybridoma Bank (Iowa)^37^. The anti-SERCA2 antibody (MA3-910) from was obtained from Affinity Bioreagents (Thermo Fisher Scientific).

### Cell culture and transfection

COS-7 and HEK-293T cells were grown in Dulbecco’s modified Eagle’s medium (Sigma) supplemented with 10% (*v/v*) foetal calf serum, 100 µg/ml penicillin and 100 µg/ml streptomycin at 37 °C in a 5% CO_2_ atmosphere. Transfections were performed using Fugene (Roche). Cells grown in 6 cm diameter dishes were transfected with 1–2 µg of plasmid DNA for immunoprecipitation experiments. For immunocytochemistry, cells were grown on 22 mm coverslips and transfected with 0.5–1 µg of DNA. Cells were grown for 24 h after transfection. Enzymatically isolated adult guinea pig ventricular cardiomyocytes (GPVCs) were prepared as described previously^59, 60^. After isolation, the GPVCs were not maintained in culture but used directly for immunocytochemistry, as described below.

### Immunoaffinity purification and mass spectrometry

Anti-myospryn (des122), -minispryn (819) and -RyR antibodies (34C) were covalently cross-linked to protein A/G-agarose as described previously^61^. Freshly dissected mouse hearts (500 mg) were homogenised in 12 ml RIPA buffer (150 mM NaCl, 50 mM Tris pH 8.0, 1% (*v/v*), Triton X-100, 0.5% (*w/v*) sodium deoxycholate) containing EDTA-free protease inhibitors (Roche) using a Polytron homogeniser operated at full speed for 20 sec. The homogenate was incubated on ice for 30 min and clarified by centrifugation at 100,000 g for 30 min in an SW41Ti rotor (Beckman). The supernatant was pre-cleared with 200 µl of protein A (Invitrogen) for 3 h at 4 °C. The pre-cleared supernatant was split into two and 50 µl of protein-A/G antibody conjugate or unconjugated protein A/G was added to each sample. IgG complexes were captured after overnight incubation at 4 °C on a blood mill by centrifugation, 1000 g for 5 min at 4 °C. The flow-through from each experiment was retained and diluted 1:1 in lithium dodecyl sulphate sample (Invitrogen) buffer containing DTT. The beads were washed twice in 1 ml of RIPA buffer containing 0.5 M NaCl and eluted in 200 µl of lithium dodecyl sulphate sample buffer containing 50 mM DTT. 30 µl samples were separated on 4–12% NuPAGE bis-TRIS gels (Invitrogen) run in MOPS/SDS running buffer (Invitrogen). Gels were fixed in 50% (v/v) methanol, 7% (v/v) acetic acid for 30 min, washed, stained with colloidal Coomassie blue stain (Gelcode Blue Stain Reagent, Pierce) for 2 h and destained in CHROMOSOLV Plus HPLC-grade water (Sigma) for 5 h. Coomassie-stained bands were manually excised and processed for mass spectrometry as described previously^62^. Briefly, proteins in the individual gel slices were digested with trypsin using a robotic gel handling system (Functional Genomics, Proteomics and Metabolomics Facility, University of Birmingham). Tryptic peptides were fractionated by liquid chromatography and analysed on a Orbitrap Velos ETD mass spectrometer (Thermo Fisher). Database searches were performed using the Mascot and SEQUEST algorithms in Proteome Discoverer™ Software (ThermoFisher).

### Immunocytochemistry

GPVCs were allowed to adhere to flamed coverslips for 30 min at room temperature, prior to fixation in 1 ml of 4% (w/v) paraformaldehyde in PBS at 4 °C for 15 min. Cells were permeabilised in 0.1% (v/v) Triton X-100 in PBS by incubation for 15 min at 4 °C. Following permeabilisation, the coverslips were washed twice in 2 ml of PBS and blocked in 10% FCS (*v/v*) in PBS for 20 min at room temperature. GPVCs were incubated with the primary antibody for 1 h at room temperature, washed twice in PBS prior to incubation for a further hour with either Alexa Fluor 488-conjugated anti-mouse IgG or Alexa Fluor 568-conjugated anti-rabbit IgG (Thermo Fisher). Coverslips were washed twice more in PBS, rinsed in molecular biology grade water and mounted on glass slides using Vectashield mounting medium (Vector Laboratories). Images were captured using a Zeiss LSM 510META confocal microscope using a 63x oil immersion objective lens and a pinhole setting of 1 Airy unit. Images were assembled using Adobe Photoshop CS4, in which they were cropped and adjusted for brightness and contrast, but not otherwise manipulated.

## Electronic supplementary material

Supplementary Information

Supplementary Dataset 1
