# Supplementary material for: Ryanodine receptors are part of the myospryn complex in cardiac muscle
Source: Sci Rep. 2017 Jul 24;7:6312. doi: 10.1038/s41598-017-06395-6 (PMC5524797; doi:10.1038/s41598-017-06395-6)
Supplement: Supplementary file 1 — Supplementary Information [file 41598_2017_6395_MOESM1_ESM.pdf]

Supplementary Information

Ryanodine receptors are part of the myospryn complex in cardiac muscle

Matthew A. Benson[1], Caroline L. Tinsley[2], Adrian J. Waite[2], Francesca A. Carlisle[2], Steve M. M. Sweet[3], Elisabeth Ehler[4], Christopher H. George[5], F. Anthony Lai[5,6], Enca Martin-Rendon[7] and Derek J. Blake[2,*]

Supplementary Figure Legends

Supplementary Figure S1. Sequence and conceptual translation of minispryn. The nucleotide sequence of mouse minispryn (clone 7E) and its conceptual translation (open reading frame, nucleotides 139 to 2289) are shown. The 6.1kb complete cDNA encodes a protein of 716 amino acid protein, minispryn. The italicised sequences correspond to dinucleotide repeats $(GA)_n$ and $(CA)_n$ in the 3'-untranslated region of the transcript. Coloured text corresponds to the amino acids forming the BBOX' (blue), BBC (green) FN3 (purple) and SPRY domains (red) in Fig. 1A.

Supplementary Figure S2. Comparative genomics of the genes encoding minispryn and myospryn. (**A**) Genomic organisation of the *Fsd2* (minispryn) and *Cmya5* (myospryn) genes in mouse. The organisation of both paralogs is remarkably similar diverging only at exons 1 and 2. Note that genes are not draw to scale. (**B**) Genomic organisation of the human and pufferfish (*Tetraodon nigroviridis*) *CMYA5* and *FSD2* gene clusters. Arrows indicate the direction of transcription. For clarity, only the *CMYA5*, *HOMER1*, *JMY* and *AP3B1* genes and their paralogs have been shaded. Abbreviations: JMY, junction-mediating and regulatory protein; *WHAMM*, WAS protein homolog associated with actin, Golgi membranes and microtubules; *SCAMP1*, secretory carrier-associated membrane protein 1); *LHFPL2*, lipoma HMGIC fusion partner-like 2; *ARSB*, arylsulfatase B; *DMGDH*, dimethylglycine dehydrogenase precursor; *BHMT2*, betaine-homocysteine methyltransferase-2; *BHMT*, betaine-homocysteine methyltransferase; *THBS4*, thrombospondin 4; *PAPD4*, PAP-associated domain-containing 4; *MTX3*, metaxin 3; *SERINC5*, serine incorporator 5; *SCARNA15*, small Cajal body-specific RNA 15.

Supplementary Figure S3. Sequence coverage of peptides derived from RyR2, myospryn and minispryn. Tryptic peptides (red) spanning the entire protein sequence

derived from RyR2, myospryn and minispryn (Table 1) are represented on the primary sequence of each protein. Proteins were purified directly from mouse heart using anti-minispryn 819-conjugated antibody beads (Fig. 3A).

Supplementary Figure S4. Immunoaffinity purification of the myospryn complex with the anti-myospryn antibody des122. (**A**) Proteins eluted from anti-myospryn-conjugated protein A beads after incubation with RIPA-extracted cardiac muscle were separated by PAGE and stained with colloidal Coomassie blue. This profile was compared to the control lane where protein A alone was incubated with the cardiac muscle extract. Unique bands were excised and processed for MS (Table 1 and Supplementary Table S1). (**B**) Western blots of fractions obtained from each immunoaffinity purification showed the specific enrichment of myospryn with the anti-myospryn beads and not with the protein A beads. Similarly, minispryn was specifically enriched with the anti-myospryn-conjugated beads. Comparison of the lysate with the flow through for each column showed that myospryn and minispryn were immune-depleted by incubation with the des122-conjugated beads. α-actinin was not detected in the eluate from the myospryn-conjugated beads despite its abundance in the starting RIPA-extracts of cardiac muscle.

Supplementary Figure S5. Immunoaffinity purification of cardiac RyRs using the 34C monoclonal antibody. Immunoaffinity purified protein complexes (10% (*v/v*)) were western blotted using the indicated antibody. 0.1% (*v/v*) of the heart RIPA extract is shown for comparison. RyRs are highly enriched in the 34C immunoaffinity purified protein mixture with both myospryn and minispryn co-purifying with the RyR. Marker sizes (M) are indicated in kDa. Asterisks represent endogenous mouse IgG purified by the protein G sepharose detected by the anti-mouse secondary antibody.

Supplementary Figure S6. Immunolocalization of minispryn and myospryn in skeletal muscle. Minispryn and myospryn were detected in longitudinal sections of guinea pig *tibialis anterior* muscle using indicated antibodies. Minispryn (819) and myospryn (des122) are found in two bands that flank the Z-line (labelled withed anti-α-actinin). This staining pattern overlaps with the distribution of the ryanodine receptors (RyR, 34C) that also flanks the Z-line. Scale bar = 20μm.

Supplementary Table S1. Mass spectrometry data for minispryn and myospryn immunoaffinity purification from mouse heart. Excel pivot table showing peptide sequences derived from RyR2, myospryn and minispryn following immunoaffinity purification of either minispryn (Fig. 3A) or myospryn (Supplementary Fig. S4) from mouse heart.

CGGGAAGGGGAGCTGGCCAGACAGAAATCCAACTTTCCAGCAGAAACCTAGGATGTTGGA
GTTAAAAGACGCATTTATGGGCCTTTAAAAAAGCAGGACTCAGAACATCCACTGACTGAG
AAGGGCTGCCCTGTGTCA**ATG**GAGGAGGAAGCAGAGGAGGAATCCGGGCTGGGCAGGTCG
M E E E A E E E S G L G R S
GCAGCCCCCAAAGACTTCCACTTTTATCACATGGACCTGTATGACTCTGAAGATAGACTG
A A P K D F H F Y H M D L Y D S E D R L
CAGCTCTTCCCAGGAGACAGCAGCAGAATCAGGAGGGAAGTAACCCAAGCCGAAGATGAC
Q L F P G D S S R I R R E V T Q A E D D
CGTGAATTAGGGGATGAATTTGTGGATGAACACAGACTGGGGACTCTGGGGTATCCTTCT
R E L G D E F V D E H R L G T L G Y P S
TACGGGATGAGGAGGAGGGACCCAGGCAGGGAGCAGAGAGACTGGGGTGAGGCAGCTGAG
Y G M R R R D P G R E Q R D W G E A A E
GCGGAGGACCTGGGCTACGGAGGAGGGCAAGGCCCTCTTGACCAGTGCCAAGACCTCCGG
A E D L G Y G G G Q G P L D Q C Q D L R
GAAGCCTACAGGTACACACATGGCCGTGCCAGCGAGGAGTATGAATGCTATGTCATCCCA
E A Y R Y T H G R A S E E Y E C Y V I P
GAGGAGGAAGACGAAGAAGAGCCTGCTGATGTCTTCTGCATCACTTGCAAAACTCCAGTA
E E E D E E E P A D V F C I T C K T P V
AGAACTGTTGAGAAGGATTTTGATACACACAAGGAACACGAAGTAACCCCAATCAGCAAA
R T V E K D F D T H K E H E V T P I S K
GCTCTGGAACATGCCAAGGATGAGGTTCACAAAAATATGTGCAAATTGGAACAGCAGATA
A L E H A K D E V H K N M C K L E Q Q I
ATTGAGATGGAAAATTTCGCTAGCCACCTGGAAGAAGTGTTCATTACTGTGGAGGAGAAT
I E M E N F A S H L E E V F I T V E E N
TTTGGAAGACAGGAACAAAACTTTGAGTGCCATTACAATGGGATCTTGGAAACCCTCGCT
F G R Q E Q N F E C H Y N G I L E T L A
CAAAAGTATGAAGAAAAAATTCAAGCTCTGGGGGAGAAAAAGAGAGAGAAACTGGAAGCC
Q K Y E E K I Q A L G E K K R E K L E A
TTATACGGACAACTGGTCAGCTGTGGAGAGAACCTCGATGCCTGCAGGGAGCTGATGGAA
L Y G Q L V S C G E N L D A C R E L M E
ACCGTAGAGGAGATGTGTCATGAGGAAAAGGTGGAGTTCTTAAAGGATGCAGTGGCTATG
T V E E M C H E E K V E F L K D A V A M
ACTGACAGACTGGGGAAATTCCTGAAAACCAAAACTGATGTGGAGCTCTCTGCACAACCT
T D R L G K F L K T K T D V E L S A Q P
GAGTTTGAAGACCAGACCTTGGACTTCTCTGATGTGGAGCAGCTGATGGACGCCATCAAT
E F E D Q T L D F S D V E Q L M D A I N
ACCATTCCAGCGCCCTCTGCTCCAGTGATAAATCCCCAGGCACCCAACTCCGCCACCGGC
T I P A P S A P V I N P Q A P N S A T G
TCCTCAGTGCGTGTATGCTGGAGCTTATATTCTGACGACACCGTGGAGAGCTACCAGCTG
S S V R V C W S L Y S D D T V E S Y Q L
TCCTATAGGCCGGTGCAGGACAGCTCATCTGGCAAGGACCGAGCAGAATTCACCATGATG
S Y R P V Q D S S S G K D R A E F T M M
GTCAAAGAAACATATTGCTCAGTGACAAATCTTGAGCCAAATACTCAGTATGAGTTTTGG
V K E T Y C S V T N L E P N T Q Y E F W
GTCATAGCTCAGAACAGGACTGGCCCCAGCCCCTGCAGTGAGCACGCAGTGTATATGACA
V I A Q N R T G P S P C S E H A V Y M T
GCCCCCTCTCCCCCCTCTATAAAAACCGAGGCAATCAGAAGCTGTGAAGAGGCTGTGCTG
A P S P P S I K T E A I R S C E E A V L
ATTTGCTGGGAGTCTGGGAACCTGAATCCGGTGGACTCCTACACGGTGGAGCTAATCCAG
I C W E S G N L N P V D S Y T V E L I Q
GCAGAGACGCCAGAAGCCTCGGGGGTAACTGAATCTGTCGTAGGCATCCCAACCTGTGAA
A E T P E A S G V T E S V V G I P T C E
TCCCTGATACAGCTGCAGCCTAGACACAGCTATACCATCTATGTGCGAGCCCTCAACGTG
S L I Q L Q P R H S Y T I Y V R A L N V
GGAGGCACCAGTGCCAGAAGTGAGCCTGCTACAGTGCACACCACAGGAAGCTACTTTCAA
G G T S A R S E P A T V H T T G S Y F Q
TTGAACAAAGACACCTGCCATCCCTGGCTGACCATTTCTGAAGATGGGTTTACGGTAGTT
L N K D T C H P W L T I S E D G F T V V
CGAAGTGAGAAGAAAAGTTTCAGAAAGGAGCTGCCGCCGAGCAAGACACAATTTACCAGG
R S E K K S F R K E L P P S K T Q F T R
TGTGTTGCTGTCATGGGGAATCTGATTCCGGTCCGAGGGCGCCATTACTGGGAAGTGGAG
C V A V M G N L I P V R G R H Y W E V E
GTGGCTGAGCATTTGGACTACACTGTGGGTGTGGCCTGTGAAGATGTCCCTAAGCAGGAA
V A E H L D Y T V G V A C E D V P K Q E
GACCTGGGAGCAAACTCCTTGTCCTGGTGCATGAGGCATACATTTGTTTCCAAAAGGCAC
D L G A N S L S W C M R H T F V S K R H
CGGTATGAGTTTCTGCACAACAAGACGACTCCAGACATCAGGATCACAGTTGCTCCGAGG
R Y E F L H N K T T P D I R I T V A P R
AAGATTGGCGTGCTGCTGGACTATGAGAACGCCAAGCTGTCCTTTTTCAATGTGGACATT
K I G V L L D Y E N A K L S F F N V D I
GCTCAGCACCTGTACACATTTAGCTGTCAGCTCCACCAGTTTGTGCATCCCTGCTTTTCC
A Q H L Y T F S C Q L H Q F V H P C F S
CTGGAAAAGTCTGGATGCCTAAAGATCTGCAATGGCATTTCAATGCCAAAGCATGTCACT
L E K S G C L K I C N G I S M P K H V T
TTCTTT**TAG**GCCCTTCTGGTATTTCCACTCTTTCTTCCTTCCTGTCCCTCAGCCACCCTC
F F *
CCCTCCCTGGCTGAACTTGGTATTCAAGGTTCCTACTGCTAGCTGAAGCTAAAGCTCACG
GTAGTGTCAGTAGATGGTGTACTGGAGCCCTTTTCACCTGTGTGCTGTGCCCTGCTGAAG
CTAGGGGGAGCCCTTATCGGGCTGTTAAATGAGGGCTCTTGTGGGGTCGAATTCTTGGAG
TGTTAAATTGCAGTTTGTGATATGTAAAGTATTTTGAGTCTCCTAAACTTATAGACCTAG
GGAGAGGGTCAGGGAAGTCATGATGGCATCAGCAGCCCCTGTACATTTGTGACATTTCAC
TGTTTACCAAGATCAGACTAGGAAAGGCAAGAAATTACAGAAAAAGAAGGAAGTCAGGTG
CCCATCTCAAGGCCATCGTGGACACTGAGTAAGAATGGTTCCCGTAGGCTCACATATTTG
AATGCTTAGTCATGGGTGGAGGCGGGGCTAGGAGTGGCACTACTTGAAAAGGATTAGACT
TATTAGGAGATGTGGCTTTGATGATCAGAATAAACCTCTCCACTACCGCTCCCATGCAGT
GAGTGCTGCCATGCTTCCTGCCATAATAAAGGGCTTAACCTCTCAAACTATAAGCCAGCC
AGCCCCCAATTTTATGTTTTCTTTTATAAGCATTATCTTGGTCATGGTGTCTCTTCACAG
AAACAGGACAATGACTAATACACAAGTTGGTTGATAACAAGGACTGGGGTATTGCTGTAA
CAGGCTCAACTGTGCTGCTTGTTGGAAGAATATGAAAGGCTTTGGAACTTTGGACTAGAA
AAGTGGCTAAGTACTTTAAGTGTGACTAATGGGAGAACAGAAGACACTGCTGAGATGATT
TGAACTGTGGGACTCAGGGGATCAAGAAGTTTAAGAGGGGATGAATATTAGTAAGTGGCC
GTTGGACCATTCTTGTGATATTTTATCAAAGAATATGGCTGCTTTTTGCCCTTGCCCTAA
AAATTTGCCTGAGGCCAAATTAAAGAGTTTTGGATTAACAGCTTTTAACAAAGGAGATTT
CAAGACAGCCTGGTATTGACCCTGCTATAAGGCTACGAGTGACTGCTCTTATTCATATCT
ATAGTGAAAAGGAGTAAGGGGAACAGGAACAGTACAAAATGTAAAGTTTGAGTAGAAAAG
GAAGACCAGGAAATGATTTGTTAGAGCCAAGTACTGGGCTTAAGGAGACAAGGTTAAAGA
AAAGCCAGGTGCTAACTGGAATAAAGGAATTGTGACCTCAGGGCAACACCACACCCCCCT
AAGCTTCCAACCTGTGAAAGGAATCAAAGGAAAGCTTGAGCAGTAAAGGAAGCCATCAAC
AGGAAGCTAGTGCAGACTGTAACTGAGAGAGGGGGTCAAGTTCCAGCCCCAGCAAGCAGA
ACTTGGCAGCTTCAGCCATGTGGTTCTTTCTGGCTTTAGAGTTAAGAATATAAGACAAGA
GGTTGTGGAATCTTCCTTCACAGTTAAAGACAGCCACTTAGTCCAGGTGTGTCAGGAGTG
TCCCTGTGTGGAGGCCCAAAAAGGATATGAAATAATTGTAAAGGTGATACCTGATTGCCT
TGGAAACCTCATGGTGTTGGAGATACCAGACACATGGGACACCTGCCAAGGACAGCTGCT
AAGAGGGTATGGAACACAC*GAGAGAGAGAGAGAGAGAGAGAGAGAGAGAGAGAGAGAGAGAG*
*AGAGAGAGAGAGAGAGAGAGAGAGAGAGCGAGAGAGAG*CGCTGCAGTCAACAAAGCTGAAAG
GAGTTGGACAGTAGACATGGAGATGCAGGACTGGAGTTAGCCTTGTTAGATTTAGGTCTT
GCTTTGGTCCAGTATTTCCTCACTATTCTCCTTTTCCTTCCTGATGGAATGGTAACTAGC
ATCTATTCTGTGCCACTGTATGTTGGAAGGATGTGATCTCTTTTTTATTTTGATCTTATG
GGTGATACAGTTAAGAGATTACCATGTATCGCAGAAGAAACTTTGGACTTGAAAACAGTG
TTAGTGTTGTGACAGCAATAGAAGAGTGACTAAGACAACCATCTATCTAGCCCAGTGTTT
CCTAACAAAAGCTGGCAGCAGCACGGCAAAATGCACACTGTTGAAACACGCTGTCTTCTG
CTTCATGGTGGTTCTAACTCCACTCTAACTCTGAAATTACGTCCAAGATGCCTAAAGGCA
GAGACTGTGTGCCTTGTTTATAACTTGACTTTGAGGGCTAACATGCCGTTAGCTGCATGC
CAATACATGCTGCCTAAAGAAGCACCTTCTTCACGCTCCACTGCTCTTGGCTTCATGTTG
CTCTTGGTGGGAAAGATGGCAGGGAGGAGAAAAATCTTAAGGGAGGCAGTGTGAAGAACA
GAAGCTACCTGTGGCTCACTGGAGAGTTACTGCCATGCCCACACACACAGTCTACTTGTG
ATTGGGGTGAATGGAGTCCCGAGGAAGGGGCTCAGATAGATGGAGATAAGAGAAAGAGCA
TTAGCTAGGATTCATCAAGGAACCATCAAGAAGTGAAAAAGCAGTGGATTTGAAGCCAAG
GACTGGGTCCAACTGCTATTTTGTATCTACTTGCCTAAAACCTACAGACCAGTGCTCCCC
ATCAAACAAAGGAAAGACTGTATTTGTCTCATAAGGATGTGATGCTTCATCTAAACCTAA
GTGCCAAGTAATGAGTGACATACTGTATATTTATGATGTTTCAAGGCAATCATCAGCTCA
CCGAAGTCAAAGCTGGGCCCTAGGGAGAGGAACGCACTGTAGCCAGCAGCTCTTGGGTGC
CCTGCCTCACTGTCTTCAGCTAAGGACCATGGTGCCTGTCTCCACCAGCTCCATTGCTGG
CTCCCCTGTTCACATTTCCAATCCCCAACTCAAGTCACTGTCAAAGCTTAGCACTCCAGC
TGCAGAGTAATAACTTCACTTGAGAAAAACCAGGACACTGTTGAGTACCAAGTCCCCTGA
TGCCGCCAACGCAGGTGTGTCCTACTCACTGCCCAGGGAGACACTCTTCTCTTTAGAATT
TTGTTTTCTAGCCCACCCTAATTCTGATGCAGTAACAACTCTCGTCTCCTTCTGGACTCT
ACTAGTGCAAGCAGCAGCTCAGATTCCTGATTTATCACTTGCCCACAGAGCTTGTCTGTC
TGTCTGTGTCTCTCTGTCTCTCTGTCTCTCTTTCT*CACACACACACACACACACACACAC*
*ACACACACACACACACACACA*ACTTCTTCTTACCCTGACATAAGGCACTGCAGATGTAAA
CACAAGCTGGCTGAAGCTGTTAATGAAATATGCATGTGTATTTAGTGAAAGAAGTTCTCT
ACTACTGAAATAGAAACCACCAAATGGGAAACGTGGATCACAGGCATGGGCAGTTTGAGT
TGTCTACTGGGATTCCTGCACTGCTTTGCACATAACACAAACATGGGGACATGACTAAAA
CATAATACCCAAAAAATTTTGATAGTTTTTTTTTTTATTTAACAAGATATCTTTGACATT
TAAAATTTACTGGAGTAAGGGATAACCAACTTCAAGCATCACAGTTACTAACCATAAAGA
ACTTTGTTTAAGCATGTTATCTTTAGGAAGAAAAATAAATCTTAAAAAAAAAAAATGAGGG
GGGAAACCCTGCTGGGTTTGTGATATATTCCAAGTCCTTATAATCCCATGTAGAAGCTGA
TGAAACTCAATTTCAAACAGGGGCAAGGTTTACCTCATGGCCATGTGCAGCTGCAACCTC
TGATAAATCTGTTAAAACACAAAACAGATAAACATTTAAAAACAAAAAAAAAAAAAAAAA
AAAAAAAAAAAAAAAAAAAAAAAAAAAA

Supplementary Figure S1

a

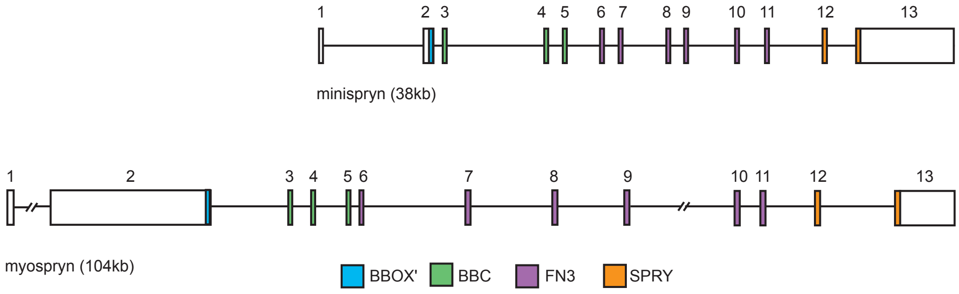

b

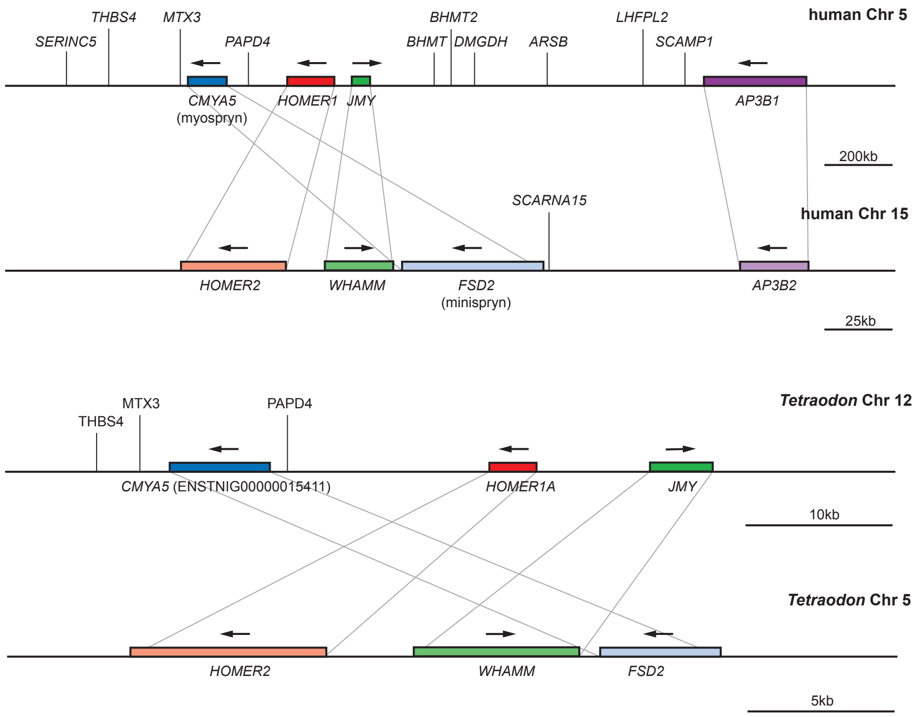

Supplementary Figure S2

RyR2
ryanodine receptor 2 [Mus musculus]
NCBI Reference Sequence:
NP_076357.2

madagegede iqflrtddev vlqctatihk eqqklclaae
gfgnr**LCFLE** **STSNSK**nvpp dlsictfvle qslsvr**ALQE**
MLANTVEKse gqvdvekwkf mmktaqgggh r**TLLYGHAIL**
LRhsysgmyl cclstsrsst dklafdvglq edttgeacww
tihpaskqrs egekvrvgdd lilvsvsser ylhlsygnss
whvdaafqqt lwsvapissg seaaqgylig gdvlrllhgh
mdecltvpsg ehgeeqr**RTV** **HYEGGAVSVH** **AR**slwrletl
r**VAWSGSHIR** wgqpfrlrhv ttgkylslme dknlllmdke
kadvk**STAFA** **FR**sskekldv gvr**KEVDGMG** **TSEIK**ygdsi
cyiqhvdtgl wltyqavdvk sarmgsiqr**K** **AIMHHEGHMD**
DGLNLSRsqh eesrtarvir **STVFLFNR**fi r**GLDALSKK**v
klptidlpie svslslqdli gyfhppdehl ehedkqnrlr
alknrqnlfq eegminlvle cidr**LHVYSS** **AAHFADVAGR**
eageswksil nslyellaal irgnrkncaq fsgsldwlis
rlerleassg ilevlhcvlv espealniik eghiksiisl
ldkhgrnhkv ldvlcslcvc hgvavr**SNQH** **LICDNLLPGR**
dlllqtrlvn hvssmrpnif lgvsegsaqy kkwyyelmvd
htepfvtaea thlrvgwast egyspypggg eewggngvgd
dlfsygfdgl hlwsgciar**T** **VSSPNQHLLR** tddvisccld
lsapsisfri ngqpvqgmfe nfnidglffp vvsfsagikv
rfllggrhge fkflpppgya acyeavlpke klkvehsrey
kqertytrdl lgptvsltqa aftpvpvdts qivlpphler
irerlaenih elwvmnkiel gwqygpvrdd nkrqhpclve
fcklpeqer**N** **YNLQMSLETL** **K**tllalgchv giadehaeek
vkkmklpkny qltsgykpap mdlsfik**LTP** **SQEAMVDKLA**
ENAHNVWARd rir**QGWTYGI** **QQDVK**nrrnp r**LVPYTLLDD**
Rtkksnkdsl reavrtllgy gyhleapdqd hasraevcsg
tgerfrifra ektyavkagr wyfefeavta gdmrvgwsrp
gcqpdlelgs ddr**AFAFDGF** **K**aqrwhqgne hygrswqagd
vvgcmvdmne htmmftlnge illddsgsel afkdfdvgdg
fipvcslgva qvgr**MNFGKD** **VSTLK**yftic glqegyepfa
vntnrditmw lskrlpqflq vpsnhehiev tr**IDGTIDSS**
PCLKvtqk**SF** **GSQNNNTDIM** **FYRLSMPIEC** **AEVFSKSVAG**
GLPGAGFYGP **K**ndledfdvd sdfevlmkta hghlvpdrid
kdketpkpef nnhkdyaqek psrlkqrfll rrtkpdystg
hsarltedvl addrddyeyl mqtstyyysv rifpgqepan
vwvgwitsdf hqydtgfdld rvr**TVTVTLG** **DEK**gkvhesi
krsncymvca gesmspgqgr nnsngleigc vvdaasgllt
fiangk**ELST** **YYQVEPSTK**l fpavfaqats pnvfqfelgr
IKNVMPLSAG **LFK**sehknpv pqcpprlhvq flshvlwsrm
pnqflkvdvs riserqgwlv qcldplqfms lhipeenrsv
dilelteqee llqfhyhtlr **LYSAVCALGN** **HR**vahalcsh
vdepqllyai enkympgllr agyydllidi hlssyatar**L**
MMNNEFIVPM **TEETK**sitlf pdenkk**HGLP** **GIGLSTSLRP**
Rmrfsspsfv sisndcyqys pefpldilka k**TIQMLTEAV**
Kegslhardp vggtteflfv pliklfytll imgifhnedl
khilqlieps vfk**EAAVPEE** **EGGTPEK**eis iedaklegee
eakggkrpk**E** **GLLQMK**lpep vklqmclllq ylcdcqvrhr
ieaivafsdd fvaklqdnqr fr**YNEVMQAL** **NMSAALTAR**k
trefrsppqe qinmllnfkd dksecpcpee irdqlldfhe
dlmthcgiel dedgsldgsn dltirgrlls lvekvtylkk
kqaekpvasd srkcsslqql isetmvrwaq esviedpelv
r**AMFVLLHRQ** **YDGIGGLVR**a lpktytingv svedtinlla
slgqirslls vr**MGKEEEKL** **MIRGLGDIMN** **NKVFYQHPNL**
MRalgmhetv mevmvnvlgg geskeitfpk mvanccr**FLC**
YFCRisrqnq kamfdhlsyl lenssvglas pamrgstpld
vaaasvmdnn elalalrepd lekvvr**YLAG** **CGLQSCQMLV**
SKgypdigwn pveger**YLDF** **LR**favfcnge sveenanvvv
rllir**RPECF** **GPALRGEGGN** **GLLAAMEEAI** **KIAEDPSRDG**
PSPTSGSSKt ldieeeeddt ihmgnaimtf yaalidllgr
capemhliha gkgeairirs ilrsliplgd lvgvisiafq
mptiakdgk**V** **VEPDMSAGFC** **PDHK**aamvlf ldrvygievq
dfllhllevg flpdlraaas ldtaalsatd malalnr**YLC**
TAVLPLLTRc aplfagtehh aslidsllht vyrlskgcsl
tkaqrdsiev cllsicgqlr psmmqhllrr **LVFDVPLLNE**
HAKmplk**LLT** **NHYER**cwkyy clpggwgnfg aaseeelhls
rklfwgifda lsqk**KYEQEL** **FK**lalpclsa vagalppdym
esnyvsmmek qssmdsegnf npqpvdtsni tipek**LEYFI**
NKyaehshdk wsmdklangw iygeiysdss k**IQPLMKPYK**
llsekekeiy rwpikeslkt mlawgwrier **TREGDSMALY**
NRtr**RISQTS** **QVSIDAAHGY** **SPRAIDMSNV** **TLSR**dlhama
emmaenyhni wakkkklele sk**GGGNHPLL** **VPYDTLTAK**e
kakdrekaqd ifkflqisgy vvsr**GFKDLD** **LDTPSIEKR**f
aysflqqlir yvdeahqyil efdggsr**SKG** **EHFPYEQEIK**
ffakvvlpli dqyfknhrly flsaasrplc tgghasnk**EK**
EMVTSLFCKl gvlvrhrisl fgndatsivn clhilgqtld
artvmktgld svksalr**AFL** **DNAAEDLEK**t menlkqgqft
htrsqpkgvt qiinyttval lpmlsslfeh igqhqfgedl
iledvqvscy r**ILTSLYALG** **TSKSIYVER**q rsalgeclaa
fagafpiafl ethldkhnvy siyntrssre raalslpanv
edvcpnipsl ek**LMTEIIEL** **AESGIR**ytqm pymmevvlpm
lcsymsrwwe hgpenhpera emcctalnse hmntllgnil
kiiynnlgid egawmkr**LAV** **FSQPIINK**vk pqllk**THFLP**
LMEKlkkk**AA** **MVVSEEDHLK** **AEAR**gdmsea ellildeftt
lardlyafyp llir**FVDYNR** akwlk**EPNPE** **AEELFR**mvae
vfiywskshn fkreeqnfvv qneinnmsfl itdtkskmsk
aaisdqerkk mkrkgdr**YSM** **QTSLIVAALK** **R**llpiglnic
apgdqelial aknr**FSLKDT** **EEEVRDIIR**s nihlqgk**LED**
PAIRwqmaly kdlpnrtedp sdpertverv lgianvlfhl
eqkskytgrg yfslvehpqr skkavwhk**LL** **SKQRK**r**AVVA**
CFRMAPLYNL **PR**hr**AVNLFL** **QGYEK**swiet eehyfedk**LI**
EDLAKPGAEL **PEEDEAMKR**v dplhqlillf srtaltekck
leedflymay adimakschd eedddgeeev ksfeekemek
qk**LLYQQAR**l hdr**GAAEMVL** **QTISASKGET** **GPMVAATLKL**
GIAILNGGNS **TVQQKMLDYL** **K**ekkdvgffq slaglmqscs
vldlnafer**Q** **NKAEGLGMVT** **EEGSGEKVLQ** **DDEFTCDLFR**
flqllceghn sdfqnylrtq tgnnttvnii istvdyllrv
qesisdfywy ysgk**DIIDEQ** **GQR**nfskaiq vakqvfntlt
eyiqgpctgn qqslahsrlw davvgflhvf ahmqmk**LSQD**
SSQIELLKEL **MDLQK**dmvvm llsmlegnvv ngtigkqmvd
mlvessnnve milk**FFDMFL** **KLKDLTSSDT** **FKEYDPDGK**g
viskrdfhka meshkhytqs etefllscae tdenetldye
efvkrfhepa kdigfnvavl ltnlsehmpn dtrlqtflel
aesvlnyfqp flgr**IEIMGS** **AK**rier**VYFE** **ISESSRTQWE**
KPQVKeskr**Q** **FIFDVVNEGG** **EKEK**melfvn fcedtifemq
laaqisesdl ner**LANKEES** **EKERPEEQAP** **R**mgffsllti
qsalfalr**YN** **VLTLVR**mlsl kslkkqmkrm kkmtvkdmvl
affssywsvf vtllhfvasv crgffrivss lllggslveg
akkikvaell anmpdptqde vrgdeeeger kplesalpse
dltdlkelte esdllsdifg ldlkreggqy kliphnpnag
lsdlmtnpvp vpevqekfqe qkakeekeek eetksepeka
egedgekeek akdekskqkl rqlhthryge pevpesafwk
KIIAYQQKLL **NYFAR**nfynm rmlalfvafa infillfykv
stssvvegke lptrtssdta k**VTNSLDSSP** **HR**iiavhyvl
eessgymept lrilailhti isffciigyy clk**VPLVIFK**
rekevarkle fdglyiteqp seddikgqwd rlvintqsfp
nnywdkfvkr kvmdk**YGEFY** **GR**dr**ISELLG** **MDKAALDFSD**
ARekkkpk**KD** **SSLSAVLNSI** **DVK**yqmwklg vvftdnsfly
lawymtmsvl ghynnfffaa hlldiamgfk tlr**TILSSVT**
HNGKqlvltv gllavvvyly tvvafnffrk fynksedgdt
pdmkcddmlt cymfhmyvgv ragggigdei edpagdeyei
yriifditff ffvivillai iqgliidafg elr**DQQEQVK**
EDMETKcfic gigndyfdtv phgfethtlq ehnlanylff
lmylinkdet ehtgqesyvw kmyqercwef fpagdcfr**KQ**
YEDQLN

myospryn (Cmya5)
stretch-responsive fibronectin protein type 3 [Mus musculus]
GenBank: CAD53474.1

```
mvavqrEDSG ITWETNSSRs stpwasgesq tsgicslegs
altsppgsvs fimdevkrtr krtqkskrgs pslrrkgskk
rnslesqdvl tnqedgpsis espvlniene kSSIGTYDKt
rrkkTASNTP PITGAIYKeh kplvlkpvyi gtvqykikmf
nsvkeelipl qfygtlpkgy vikeihyrrg kdssislepd
lsnggsnivp qrKLAQSPEE DKVRelappw rgalskgsrT
SLFSHEEQKK TYADSNLNVP SSTEHAFPSS ARNDTADQEE
NLSLPQMMPQ QPADESKthr MEPPSIPATM VLERAKEELE
QNAQGKesse ddasvltgsa ddvqqeglvs vnhsmpweae
kesletgppr papaiqekFE PDMEGLEPIS TEKTEQASEY
VTSSEPIVHR EEEHAPEPIV HREEEHAPEP IVHREEEHAP
EPIVHReeeh apesivhree ehapesivqr eeehapepes
ivhreeehap gpvpivhree ehvpelesiv hrGEEHAPEP
IVHRDEGHAP EPIVHReeeh vpepivhrDE GHAPEPIVHR
eeehvpepes ivhreeehap epmvlrkaql ergvetstpi
tdttepedss leeeiieldy pesplaskET SPSPLSPEVE
HRKEPILPTQ MTFTPERitl seeereenes vstdsafvse
ysvlqdlnht pekleveavs vsdvkssnep avfseddeer
esyspamtsv seqslspstt ektsaiqspl fstvspvlsg
deasesvchs pesesaaeys vpahaqelll kTGDHKLPLK
sqrvsepiiq aedekedigl lppaalsqav lsedeslgsg
sfasdskLPF KPSVSQNATR espqkTIDDM PQFKPRGLSD
PATLLEEEKe aigvglsssn evsavecalp pqttellses
happpwaiss eqvvqseegs rdqqrGSFSS TPELGHTSPL
LLKgassptg lseqgqeedn igplspdsaf asefsfspyp
tqelekrelg rdsplcltsp seqtvlsded teeadlfspd
sasqvsippy rIAETEQNKV EPDELLPRrs apdypyfsea
deeeagssvv tpvpehseps qereesspcr pvfedlsllp
sadktgqaet msdvptists vseylilaqq ektqaslepe
aedlvpppts gwekrdakss lpavtiaass salssvvkee
ttsvlptsqp svspestcvl kpeqeptapl tltsadeqma
lprvgrEKAV LDSQEATAHK SQDQTPEPRL PNVPGSGMKY
SVLSDLGDEP KADVKLNLAP TVTSELEQRm lskNEPEVAK
PHSPPEETSI SGPKvlsavk tevkqeskit rELPAASSGR
ergaehsppa ppalpalmee mgkdteasss attvpvtkld
snstkLGRDE VLTDPSLASP VEHPGLKgvg kSELGSGLPL
PSMSTSEVLR PEPKlpvnsg vevereднep pplqvsptsk
ptvpndkhee itrspdsenv vsddlaptll alrhemnrqa
eetsspvpgs flsgeqelik LPPEPEKhkQ LSEVPTAGSE
LIDSRdrdrS LGIEPVKPIG TEPGPSILEK GPAELQRrgk
eqeenrklpv pasapletas fdlpieqkep krtlhegqav
evpdessssa dkpelgvkql aekKENLEQP KPFVTTERas
vtgskVKESL ISPKdntwml ekpdglvnqh edrKPGTGQL
ESSESTDLMS EKLGAAPLDT DHTSETRNQE TSKAPVSGEK
lsqeprrvqs kavddseegr KLASGNVEVL TQSKsvpavk
akATPQPPET PEVTQKPSEK SLVTEQGLPA EKgkkgissf
kswmssllfg ssipdskVSD NEDLETRPGP SVEKAVPAIE
PKgtvpaevn iaekpavhsl pevtvklaee pkgvsvkSSI
SQDLKEKLTF LSNEDVLKQP Ksnsenygqk elpgfsegmg
eslatsvgdk hpgihpcspm gekVGMEEAQ NMAPLHITES
QRrqkpevss psmwnisark eepssdhket wlsssdvvdr
mpqkpkSAQS AFTRMNSEEP ASMILPVESK GSLSDLGEDR
LRqempkpts lehceeever pteekdgwet rSFSLAGKRG
LAEKQEIMAP LELRENEAVG ELQRMPESRP FKLEESKaae
rleqrispte klmekpsktl aldrrekevq ewvfsegekq
eyppaampvp gasavsldka qphllakptp vvekpehivt
evypeirerK AAETQPHPQE EGKTLVEKtk vsrVESPHGE
ETDGHSLTQE GNLELEKsge srvdlkeerr rfvmpelslg
asvaaedgsv qprplskdaa rvsdmtdetk hlgtpptqps
avepqtlvlg tsvehtvkkq etwsdrptvh tfqtskDYTE
EMLKQSVLIS KHHLEAVEDV HRneppssaa snytqfmlsa
seisadgvpp mggtaqepeg tsvkdeefsv tskpaglsed
qksafsiise gceilnihap afipsvdqee seqmqdkLQY
LEEKasfksi svhdekkaaa shktqkSKLE VPDRKITSLK
ENETKethkt keeiatdsgm gdftpiqptv sgeedyfekY
TLIDYNLSPG SGKQKSTVEE SSEEATKtlt sfpessaeqa
ldheynlvkL DESFYGPEKD DSKlshaemq kSLAIQKPDD
Rnapkgisrd vdsrSPGMPL FDVEEGVLSK RQIFPTTPKa
vnpelleepp alsffykdly egaggekneg etasegdsvd
setsfprrhs dtddgpgmyf ekyilkddil hdetvtqedq
gqgleekpvg eedsqqlrva ereirRKPET SFWEKnleeq
hkvvgrEGEP TGHMETLDEA AMQQKAPITE QVRavtqkms
yavpfqdtrc vlesepssqg neagnaspdv nlnvpvqvsf
peeesaagat yapevlqerL VPSVSREERl hntpvqdeyd
fvgslnqeaa sqailpeepg sesspkEVLS QGSESFEHIR
EQELTSEGEP RMSASQEVWD RTEDQSARes vtaktqkepk
KTQAESYCYT CKSLVSEMDK aldihkDHEV SALDTAISAV
KVQLGEFLEN LQEKslriea fvseiesffn tieekcskne
kRLEMQNEEM MKRVLAQYDE KAQSFEEVKk kkmeflhdqm
vhflqsmdta kDTLETIVRg akeldetvfl asfeeinerl
lsamestasl enmpaafslf ehyddssars dqmlkQVAVP
QPPRlepqep ssatsttiav ywsvnkedvv dsfqvycvee
pqddqeinel veeyrltvkE SCCIFEDLEP DRcyqvwvma
vnftgcslps eraifrtaps tavihvedct vcwntatvrw
rpanpeatet ytleycrqhs pegeglrsfs gikglqlkvn
lppndnyffy vrATNASGTS EQSEAALIST Rgtrflllre
tahpalqisa ngtvisfser rrlteipsvl geelpacgqh
ywettvadsp ayrLGICTSS AVRagalgqg etswymhcse
pqrytffysg ivsevhater parVGILLDY TNQRllfina
esgqllfivr hrFNEGVHPA FALEKPGRCT LHLGLEPPDS
VRhk
```

minispryn (Fsd2)
fibronectin type III and SPRY domain-containing protein 2 [Mus musculus]
NCBI Reference Sequence: NP_766492.2

```
meeeaeeesg lgrsaapkDF HFYHMDLYDS EDRLQLFPGD
SSRirREVTQ AEDDRELGDE FVDEHRLGTL GYPSYGMRrr
dpgreprdwg eaaeaedlgy gggqgpldqc qdlreayryt
hgraseeyec yvipeeedee epadvfcitc ktpvrTVEKD
FDTHKEHEVT PISKALEHAK DEVHKNMCKl eqqiiemenf
ashleevfit veenfgrqeq nfechyngil etlaqkYEEK
IQALGEKkre klealygqlv scgenldacr ELMETVEEMC
HEEKVEFLKD AVAMTDRlgk flktktdvel saqpefedqt
ldfsdveqlm daintipaps apvinpqapn satgssvrvc
wslysddtve syqlsyrpvq dsssgkDRAE FTMMVKetyc
svtnlepntq yefwviaqnr TGPSPCSEHA VYMTAPSPPS
IKteairsce eavlicwesg nlnpvdsytv eliqaetpea
sgvtesvvgi ptcesliqlq prHSYTIYVR ALNVGGTSAR
SEPATVHTTG SYFQLNKdtc hpwltisedg ftvvrsekks
frKELPPSKt qftrCVAVMG NLIPVRgrhy wevevaehld
ytvgvacedv pkQEDLGANS LSWCMRhtfv skrHRYEFLH
NKmtpdirit vaprKIGVLL DYENAKlsff nvdiaqhlyt
fscqlhqfvh pcfsleksgc lkICNGISMP Khvtff
```

Supplementary Figure S3

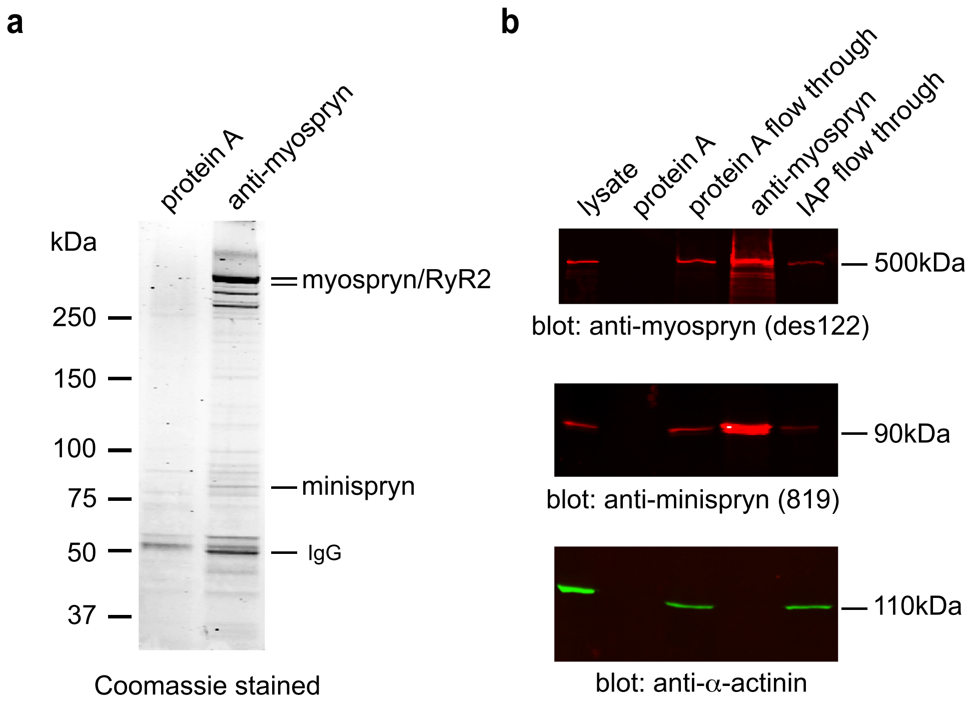

Supplementary Figure S4

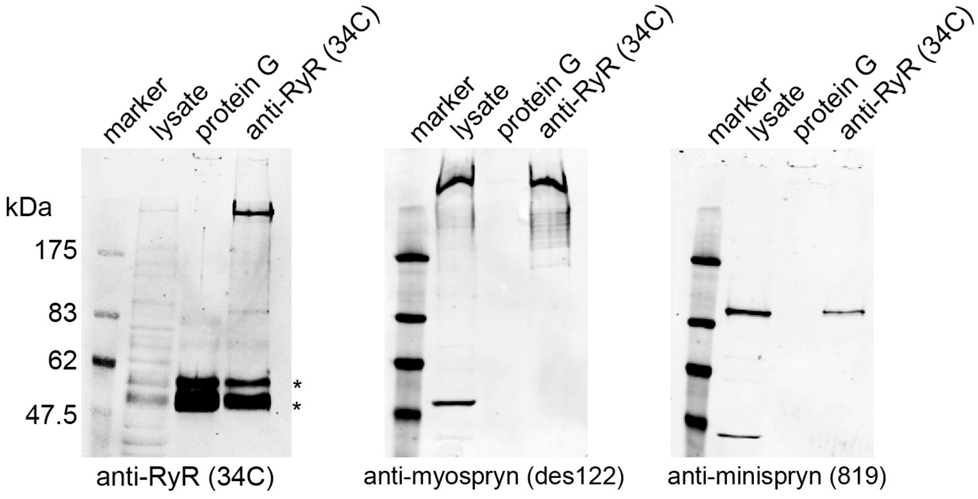

Supplementary Figure S5

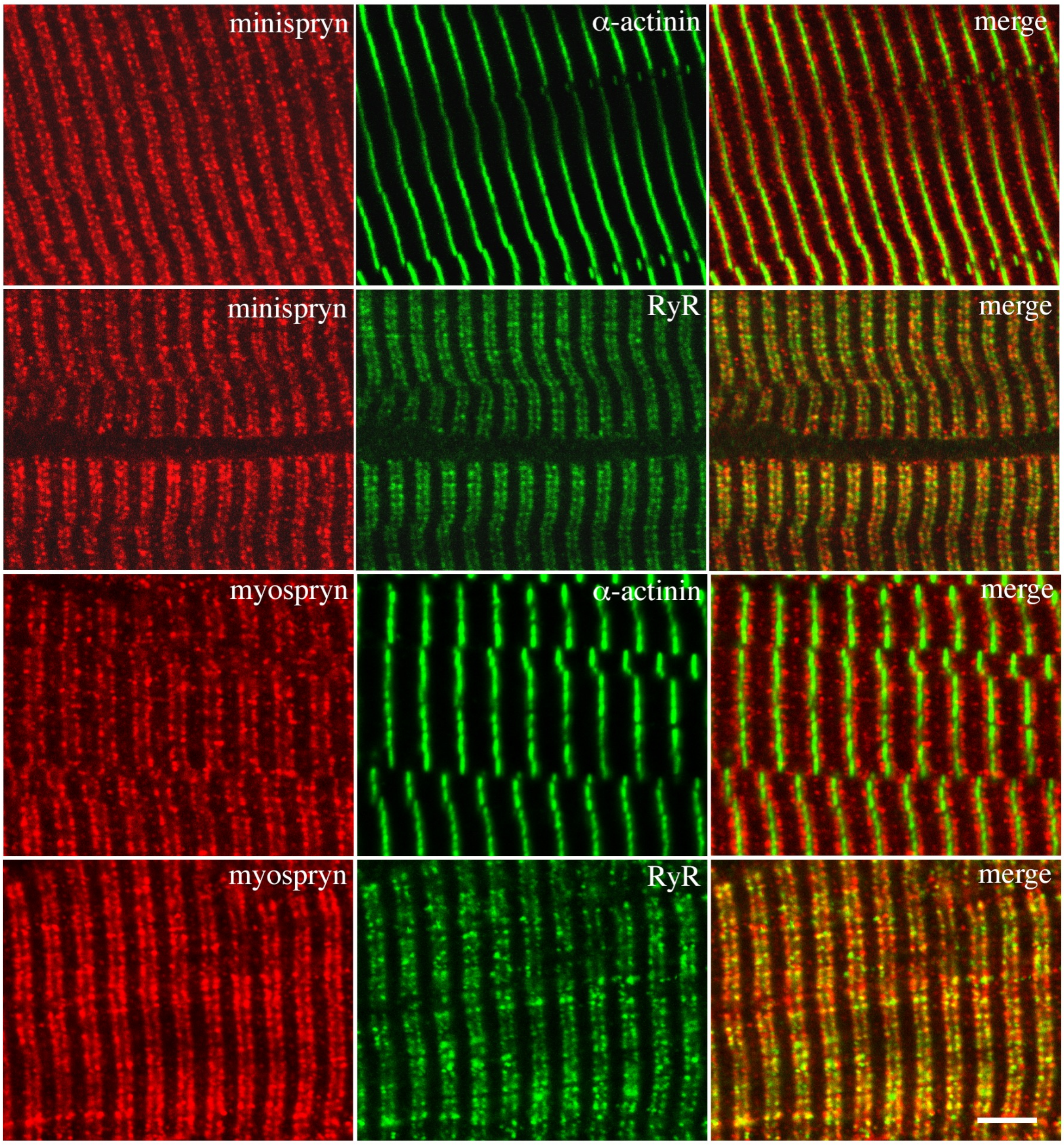

Supplementary Figure S6
